# Potential effects of climate change on dengue transmission dynamics in Korea

**DOI:** 10.1371/journal.pone.0199205

**Published:** 2018-06-28

**Authors:** Hyojung Lee, Jung Eun Kim, Sunmi Lee, Chang Hyeong Lee

**Affiliations:** 1 Graduate School of Medicine, Hokkaido University, Sapporo, Japan; 2 Department of Mathematical Sciences, Ulsan National Institute of Science and Technology, Ulsan, Republic of Korea; 3 Department of Applied Mathematics, Kyung Hee University, Yongin, Republic of Korea; University of Malaya, MALAYSIA

## Abstract

Dengue fever is a major international public health concern, with more than 55% of the world population at risk of infection. Recent climate changes related to global warming have increased the potential risk of domestic outbreaks of dengue in Korea. In this study, we develop a two-strain dengue model associated with climate-dependent parameters based on Representative Concentration Pathway (RCP) scenarios provided by the Korea Meteorological Administration. We assess the potential risks of dengue outbreaks by means of the vector capacity and intensity under various RCP scenarios. A sensitivity analysis of the temperature-dependent parameters is performed to explore the effects of climate change on dengue transmission dynamics. Our results demonstrate that a higher temperature significantly enhances the potential threat of domestic dengue outbreaks in Korea. Furthermore, we investigate the effects of countermeasures on the cumulative incidence of humans and vectors. The current main control measures (comprising only travel restrictions) for infected humans in Korea are not as effective as combined control measures (travel restrictions and vector control), dramatically reducing the possibilities of dengue outbreaks.

## Introduction

Dengue fever is a mosquito-borne viral disease transmitted by *Aedes* mosquitoes. Dengue is endemic in more than 100 countries, including African, American, Asian, and Western Pacific countries with tropical climates. Dengue virus includes four serotypes (DEN 1-4), and DEN 2 and DEN 3 are prevalent in tropical countries [1]. Infection with one serotype confers permanent immunity to that serotype, as well as temporary cross-immunity to other serotypes. Furthermore, people reinfected by other serotypes are at risk of developing more serious diseases such as dengue hemorrhagic fever (DHF) and dengue shock syndrome (DSS) [2–4]. Every year in South Korea, it is reported that a few hundred people are infected during travel to dengue-endemic countries. However, there have been no domestic infections reported so far [5]. Nevertheless, with a gradual change toward a subtropical climate owing to global warming, Korea could face a spread of domestic dengue in the near future [6, 7].

Climate factors such as temperature and precipitation significantly affect the life cycle of dengue mosquitoes [8–15]; thus, these factors need to be included in mathematical models of dengue transmission. Many studies have investigated the effects of climate factors on dengue transmission. Climate change impacts factors relevant to the mosquito population. In particular, the temperature has a strong influence on dengue transmission and the *Aedes* mosquito population [8–10]. Chen and Hsieh investigated the impact of temperature variation on dengue transmission dynamics in a single-strain model [11]. A susceptible-exposed-infectious-recovered (SEIR) model with four dengue strains has been employed to study the seasonal population dynamics of mosquitoes [12]. Dengue incidences in Thailand, Taiwan, Singapore, and Brazil are associated with seasonal patterns in temperature, relative humidity, and rainfall [11–14]. Statistical approaches have revealed that these seasonal patterns play a significant role in dengue transmission [8, 16]. A two-patch dengue transmission model incorporating seasonality has been employed to explore the impact of different patch-specific control strategies [17]. In [18, 19], the temperature-dependent parameters obtained from laboratory data ranged from 10°*C* to 37°*C*. Moreover, the amount of rainfall may affect the larval population size [15], and the rainfall pattern has a certain effect on the larval density [20]. A recent study by ten Bosch et al. considered six different dengue models, with important dengue characteristics such as cross-immunity, antibody-dependent enhancement and seasonal forcing [21]. The authors adopted a pattern-oriented modeling strategy to capture dengue dynamics such as multi-annual fluctuations and the mean duration between peaks.

In South Korea, 1,331 cases of dengue fever were reported in the period 2010–2016 [5], and all infected people were travelers who returned from endemic countries such as Thailand, Philippines, Vietnam, and Indonesia [5, 22]. Several studies demonstrated that the risk of an autochthonous dengue outbreak increases as international travel to endemic areas increases [23, 24]. Because *Aedes* mosquitoes have been found on Jeju Island, which has a humid subtropical climate and is warmer than other regions in Korea [25, 26], and the inflow of travelers to the island has recently increased [27], the potential for autochthonous dengue transmission on Jeju Island is greater than in other regions of Korea. Therefore, we formulate single-strain and two-strain dengue transmission models with a focus on Jeju Island, and explore the impact of climate change on the dengue transmission dynamics under Representative Concentration Pathway (RCP) scenarios. Furthermore, we assess the potential risks of dengue outbreaks via the vectorial capacity and intensity, and investigate the effects of control measures for infected humans and vectors.

## Methods

In this section, we develop single-strain and two-strain dengue transmission models using a system of nonlinear differential equations.

### Single-strain dengue transmission model

**Fig 1:**
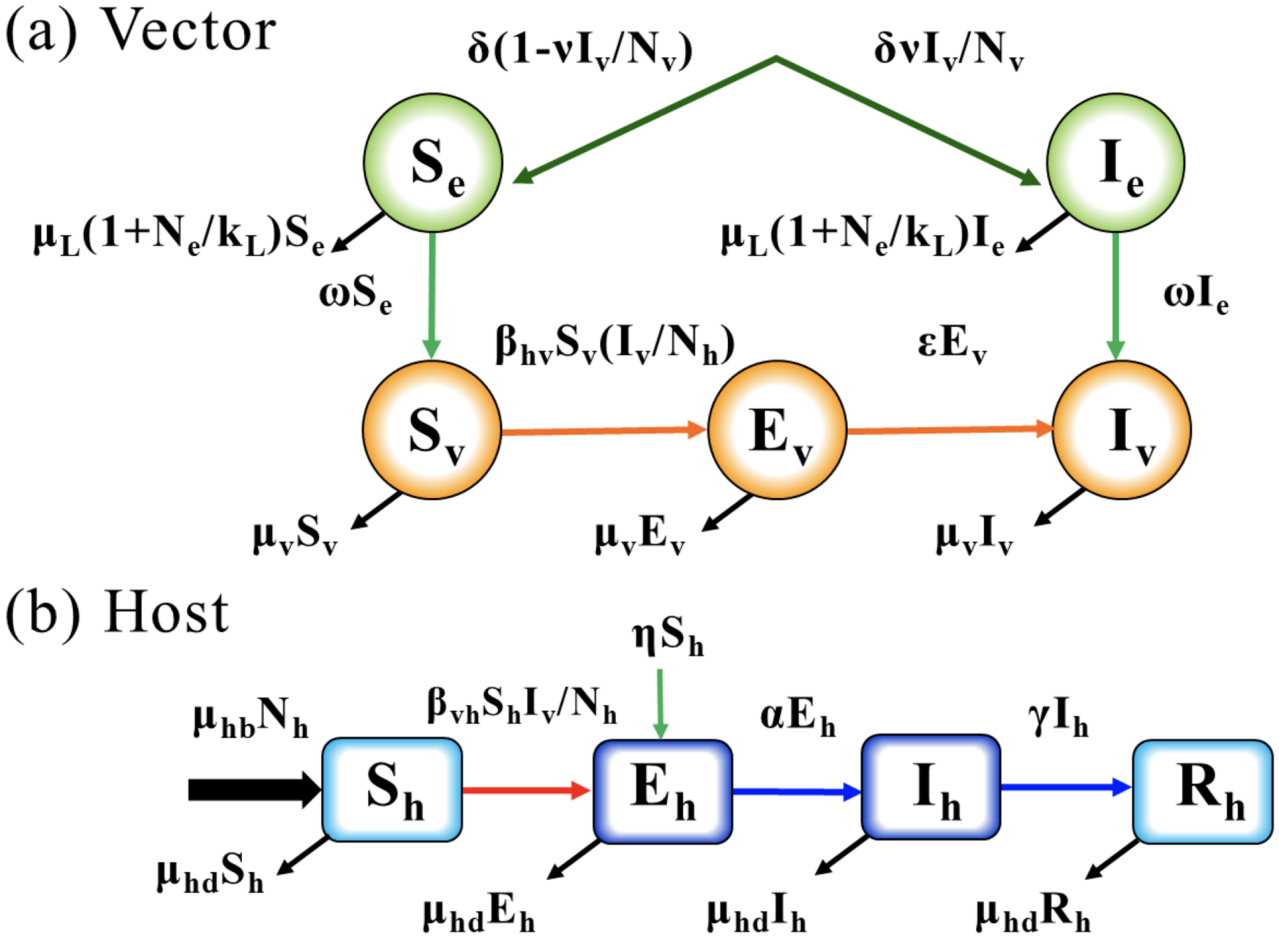
Single-strain dengue transmission model.

The single-strain model includes various states of mosquito larvae (classes that are susceptible (*S*_*e*_) and infectious (*I*_*e*_) by vertical infection), female adult mosquitoes (susceptible (*S*_*v*_), infected but not infectious (*E*_*v*_), and infectious (*I*_*v*_)), and the humans (susceptible (*S*_*h*_), infected and not infectious (*E*_*h*_), infectious (*I*_*h*_), and recovered (*R*_*h*_)). The total larva population, female adult mosquito population, and total human population are denoted by *N*_*e*_, *N*_*v*_, and *N*_*h*_, such that *N*_*e*_ = *S*_*e*_ + *I*_*e*_, *N*_*v*_ = *S*_*v*_ + *E*_*v*_ + *I*_*v*_, and *N*_*h*_ = *S*_*h*_ + *E*_*h*_ + *I*_*h*_ + *R*_*h*_. A schematic diagram of this model is presented in Fig 1.

The dynamics of the model are described by a system consisting of nine ordinary differential equations, shown in (1):

$$\begin{array}{ccc} \frac{dS_{e}}{dt} & = & \delta (1-\nu I_{v}/N_{v})-\omega S_{e}-\mu_{l}S_{e}\\ \frac{dI_{e}}{dt} & = & \delta \nu I_{v}/N_{v}-\omega I_{e}-\mu_{l}I_{e}\\ \frac{dS_{v}}{dt} & = & \omega S_{e}-\beta_{hv}S_{v}I_{h}/N_{h}-\mu_{v}S_{v}\\ \frac{dE_{v}}{dt} & = & \beta_{hv}S_{v}I_{h}/N_{h}-\varepsilon E_{v}-\mu_{v}E_{v}\\ \frac{dI_{v}}{dt} & = & \varepsilon E_{v}+\omega I_{e}-\mu_{v}I_{v}\\ \frac{dS_{h}}{dt} & = & \mu_{hb}N_{h}-\beta_{vh}S_{h}I_{v}/N_{h}-\eta S_{h}-\mu_{hd}S_{h}\\ \frac{dE_{h}}{dt} & = & \beta_{vh}S_{h}I_{v}/N_{h}+\eta S_{h}-\alpha E_{h}-\mu_{hd}E_{h}\\ \frac{dI_{h}}{dt} & = & \alpha E_{h}-\gamma I_{h}-\mu_{hd}I_{h}\\ \frac{dR_{h}}{dt} & = & \gamma I_{h}-\mu_{hd}R_{h}. \end{array}$$ (1)

In the system (1), the parameters related to larvae and mosquitoes include the number *δ* of new recruits in the larva stage, the maturation rate *ω* of pre-adult mosquitos, the mortality rate *μ*_*v*_ of adult mosquitos, and the mortality rate *μ*_*l*_ of larvae, defined by *μ*_*l*_ = *μ*_0_(1 + *N*_*e*_/*k*_*l*_), where *μ*_0_ is the minimum mortality rate and *k*_*l*_ is the carrying capacity of larvae in the environment [15]. Here, 1/*ε* refers to the extrinsic incubation period, and *ν* represents the rate of vertical infection from infected mosquitoes to eggs. The parameters *b*_*m*_ and *b*_*h*_ represent the probability of infection (human to mosquito) per bite and the probability of infection (mosquito to human) per bite, respectively, *β*_*vh*_ = *x*_1_*bb*_*h*_ is the transmissible rate from mosquito to human, and *β*_*hv*_ = *x*_2_*bb*_*m*_ is transmissible rate from human to mosquito, where *b* is the daily biting rate of a mosquito and *x*_1_ and *x*_2_ are the transmission probabilities, which can be obtained by data fitting. The parameters *μ*_*hb*_ and *μ*_*hd*_ represent the human birth rate and death rate, respectively, and 1/*α* and 1/*γ* are the latent period and infectious period for humans, respectively. The inflow rate of infection due to international travelers is defined by *η*.

The seasonal reproduction number of the system (1) at time *t* in the absence of the inflow rate of international travelers (i.e., *η* = 0) is given by the next generation matrix, as follows (for a detailed derivation, see Section A in S1 Appendix):

$$\begin{array}{ccc} R_{s} & = & \frac{A}{2}+\frac{1}{2}\sqrt{A^{2}+4\Lambda }\\ A & = & \frac{\delta \left(t\right)k_{l}\left(t\right)\omega \left(t\right)\nu }{\mu_{v}\left(t\right)N_{v}\left(t\right)\left(k_{l}\left(t\right)\left(\mu_{0}+\omega \left(t\right)\right)+\mu_{0}S_{e}\left(t\right)\right)},\\ \Lambda & = & \frac{\alpha \beta_{hv}\left(t\right)\beta_{vh}\left(t\right)\varepsilon \left(t\right)S_{h}\left(t\right)S_{v}\left(t\right)}{\left(\alpha +\mu_{hd}\right)\mu_{v}\left(t\right)\left(\varepsilon \left(t\right)+\mu_{v}\left(t\right)\right)\left(\mu_{hd}+\gamma \right)N_{h}{\left(t\right)}^{2}} \end{array}$$

### Two-strain dengue transmission model

**Fig 2:**
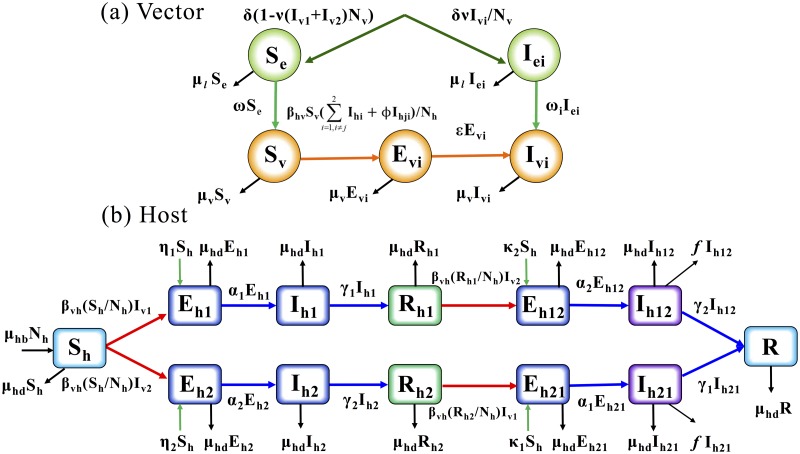
Two-strain dengue transmission model.

Dengue hemorrhagic fever (DHF) and dengue shock syndrome (DSS) affect possible reinfections with another serotype, owing to the effects of antibody-dependent enhancement (ADE) [28]. In the two-strain model, we distinguish two strains as the major strain 1 and minor strain 2, such that the human population consists of 12 compartments: susceptible (*S*_*h*_), exposed to strain *i* (*E*_*hi*_), infectious with strain *i* (*I*_*hi*_), recovered from infection with strain *i* (*R*_*hi*_), exposed (*I*_*hij*_) to strain *j* from *R*_*hi*_, infectious (*I*_*hij*_) with strain *j* from *R*_*hi*_, and finally the recovered and life-long immune population against the two strains (*R*). Thus, the total human population is *N*_*h*_ = *S*_*h*_ + *E*_*h*1_ + *E*_*h*2_ + *I*_*h*1_ + *I*_*h*2_ + *R*_*h*1_ + *R*_*h*2_ + *E*_*h*12_ + *E*_*h*21_ + *I*_*h*12_ + *I*_*h*21_ + *R*. The female mosquito population, denoted by *N*_*v*_, is divided into five compartments: susceptible *S*_*v*_, infected but not infectious *E*_*vi*_, and infectious *I*_*vi*_ for strain *i*. Thus, *N*_*v*_ = *S*_*v*_ + *E*_*v*1_ + *E*_*v*2_ + *I*_*v*1_ + *I*_*v*2_. Similarly, the larva population is denoted by *N*_*e*_ = *S*_*e*_ + *I*_*e*1_ + *I*_*e*2_. The full diagram of the dengue transmission model with two strains is presented in Fig 2.

The model is described by a system of 21 differential equations in (2) and (3). In this model, the parameters 1/*α*_*i*_ and 1/*γ*_*i*_ represent the latent and infectious periods for humans with strain *i*, respectively. The ADE factor *ϕ* is the rate contributing to the force of secondary infection. The parameter *ω*_*i*_ denotes the maturation rate of a pre-adult mosquito for each strain *i*, where *ω*_1_ + *ω*_2_ = *ω*. We assume that *ω*_*i*_ = 0.5*ω*. The inflow rate of infected international travelers with the primary (secondary) infection is denoted by *η*_*i*_ (*κ*_*i*_) for strain *i*. Furthermore, *f* represents the fatality rate for the secondary infection, and deaths by the secondary infection are represented by *D* in (3).

$$\begin{array}{ccc} \text{Vector}\\ \frac{dS_{e}}{dt} & = & \delta (1-\nu \sum_{i=1}^{2}I_{vi}/N_{v})-\left(\omega_{1}+\omega_{2}\right)S_{e}-\mu_{l}S_{e}\\ \frac{dI_{ei}}{dt} & = & \delta \nu \frac{I_{vi}}{N_{v}}-\omega_{i}I_{ei}-\mu_{l}I_{ei}\\ \frac{dS_{v}}{dt} & = & \left(\omega_{1}+\omega_{2}\right)S_{e}-S_{v}\sum_{i=1,i\neq j}^{2}\beta_{hv}\left(I_{hi}+\phi I_{hji}\right)/N_{h}-\mu_{v}S_{v}\\ \frac{dE_{vi}}{dt} & = & \beta_{hv}S_{v}(I_{hi}+\phi I_{hji})/N_{h}-\varepsilon E_{vi}-\mu_{v}E_{vi}\\ \frac{dI_{vi}}{dt} & = & \omega_{i}I_{ei}+\varepsilon E_{vi}-\mu_{v}I_{vi} \end{array}$$ (2)

$$\begin{array}{ccc} \text{Host}\\ \frac{dS_{h}}{dt} & = & \mu_{hb}N_{h}-S_{h}\sum_{i=1}^{2}\beta_{vh}I_{vi}/N_{h}-\sum_{i=1}^{2}\left(\eta_{i}+\kappa_{i}\right)S_{h}-\mu_{hd}S_{h}\\ \frac{dE_{hi}}{dt} & = & \beta_{vh}S_{h}I_{vi}/N_{h}+\eta_{i}S_{h}-\left(\alpha_{i}+\mu_{hd}\right)E_{hi}\\ \frac{dI_{hi}}{dt} & = & \alpha_{i}E_{hi}-\gamma_{i}I_{hi}-\mu_{hd}I_{hi}\\ \frac{dR_{hi}}{dt} & = & \gamma_{i}I_{hi}-\beta_{vh}R_{hi}I_{vj}/N_{h}-\mu_{hd}R_{hi}\\ \frac{dE_{hij}}{dt} & = & \beta_{vh}R_{hi}I_{vj}/N_{h}+\kappa_{j}S_{h}-\alpha_{j}E_{hij}-\mu_{hd}E_{hij}\\ \frac{dI_{hij}}{dt} & = & \alpha_{j}E_{hij}-(\gamma_{j}+\mu_{hd}+f)I_{hij}\\ \frac{dR}{dt} & = & \left(\gamma_{2}I_{h12}+\gamma_{1}I_{h21}\right)-\mu_{hd}R\\ \frac{dD}{dt} & = & f(I_{h12}+I_{h21}) \end{array}$$ (3)

The seasonal reproduction number for the systems (2) and (3) in the absence of the inflow of international travelers (i.e., *η*_*i*_ = *κ*_*i*_ = 0) is given by the next generation matrix, as follows (for the derivation, see Section A in S1 Appendix):

$$\begin{array}{ccc} R_{s} & = & max(R_{s1},R_{s2})\\ R_{s1} & = & \frac{A}{2}+\frac{1}{2}\sqrt{A^{2}+4\Lambda_{1}},\;\;R_{s2}=\frac{A}{2}+\frac{1}{2}\sqrt{A^{2}+4\Lambda_{2}}\\ \text{where}\;\;\;A & = & \frac{\delta \left(t\right)k_{l}\left(t\right)\omega_{i}\left(t\right)\nu }{\mu_{v}\left(t\right)N_{v}\left(t\right)\left(k_{l}\left(t\right)\left(\mu_{0}+\omega_{i}\left(t\right)\right)+\mu_{0}S_{e}\left(t\right)\right)},\\ \Lambda_{i} & = & \frac{\alpha_{i}\beta_{hv}\left(t\right)\beta_{vh}\left(t\right)\varepsilon \left(t\right)S_{h}\left(t\right)S_{v}\left(t\right)}{\left(\alpha_{i}+\mu_{hd}\right)\mu_{v}\left(t\right)\left(\varepsilon \left(t\right)+\mu_{v}\left(t\right)\right)\left(\mu_{hd}+\gamma_{i}\right)N_{h}{\left(t\right)}^{2}},\;\;\text{for}\;i=1,2. \end{array}$$

### RCP scenarios

**Fig 3:**
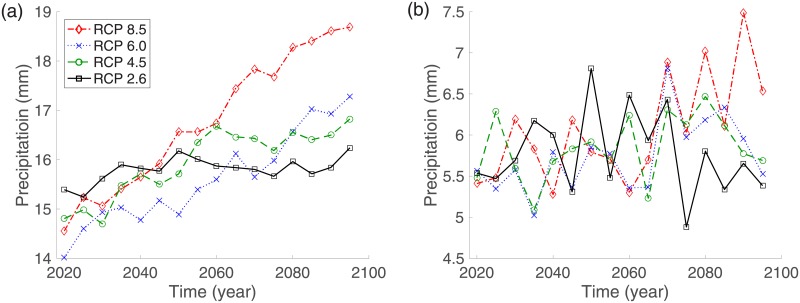
Temperature based on RCP 2.6, RCP 4.5, RCP 6.0, and RCP 8.5 climate change scenarios on Jeju Island: (a) the five-year average daily temperature from 2020 to 2099, and (b) the five-year average daily precipitation from 2020 to 2099.

For its fifth Assessment Report in 2014, the Intergovernmental Panel on Climate Change (IPCC) developed four greenhouse gas concentration trajectories (Representative Concentration Pathways (RCPs)) to facilitate future assessments of climate change, including emissions mitigation. RCPs are named according to the radiative forcing levels from 2.6 to 8.5 *W*/*m*^2^ shown in Table 1. These comprise the lowest forcing level scenario (RCP 2.6), two medium stabilization scenarios (RCP 4.5/RCP 6.0), and the high-end baseline emission scenario (RCP 8.5). The Korea Meteorological Administration (KMA) provides future climate data generated from RCP scenarios [29]. We compare the dynamics of dengue prevalence between the four types of climate change scenario RCP 2.6, 4.5, 6.0, and 8.5 to investigate the effect of climate change on dengue outbreaks. Temperature and precipitation data for Jeju Island under the assumption of the RCP scenarios consist of the average values estimated for the four regions Jeju, Seoguipo, Sungsan, and Gosan, which are located in a warm temperate zone [29]. While the climate zones of Jeju Island are divided into a warm temperate zone, grassland zone, and cool temperature zone according to the altitude, more than 98% of the population of Jeju Island lives in the warm temperate area, which is less than 200 *m* above sea level [30]. Thus, our model focuses on the warm temperate area using the climate data provided by KMA. Fig 3(a) illustrates the average daily temperature for five year intervals from 2020 to 2099. According to the RCP 4.5 and RCP 8.5 scenarios, the average temperature on Jeju Island in Korea will increase by 2.02°*C* in RCP 4.5 and 4.13°*C* in RCP 8.5 over 80 years. Fig 3(b) illustrates the average daily precipitation for five year intervals on Jeju Island from 2020 to 2099.

**Table 1:** Representative Concentration Pathway scenarios.

Scenarios	Description	*CO*_2_ (ppm)
RCP 2.6	Peak in radiative forcing at ∼ 3*W*/*m* before year 2100 and decline	420
RCP 4.5	Stabilization without an overshoot pathway to ∼ 4.5*W*/*m* at stabilization after year 2100	540
RCP 6.0	Stabilization without an overshoot pathway to ∼ 6*W*/*m* at stabilization after year 2100	670
RCP 8.5	Rising radiative forcing pathway leading to 8.5 *W*/*m* in year 2100	940

### Parameter estimation

#### Climate-independent parameters

No indigenous dengue cases have been reported in Korea. All those diagnosed in Korea have returned after visiting to an endemic area. The dengue virus can invade new areas with the potential for risks. Global warming and globalization effects such as increased international travel, trade, and transportation affect the spread of dengue proliferation [24, 31, 32]. Jeju Island is located at the southern end of Korea, which has a borderline subtropical climate, and the larvae of the *Aedes albopictus* mosquito have been found on Jeju Island since 2010, which suggests that a dengue outbreak could occur on Jeju Island in the near future. Increased international travel and climate change could be important factors in dengue fever outbreaks in Korean territory, including Jeju Island. The Korea Centers for Disease Control and Prevention (KCDC) provided annual reports between 2001 and 2016 and monthly reports between 2012 and 2016 on cases of dengue fever [5]. According to the data from the KCDC, the number of infected international travelers has gradually increased since 2001. We estimate the inflow rate for each year between 2020 and 2070 by fitting the annual data in Table 2 with the logistic function 897.9035 × (1 + *e*^−0.2398(*x*−2018.8)^)^−1^ for the year *x*, and compute the daily inflow rate by interpolating the monthly data in Table 3. The daily inflow rate, or daily new infection rate by immigration, is denoted by *η* in the single-strain model (1) and *η*_*i*_, *κ*_*i*_ in the two-strain model (3). Let *η*_*i*_ = *ξ*_*i*_*η*, *κ*_*i*_ = *τ*_*i*_*η*, where *ξ*_*i*_, *τ*_*i*_ are weights satisfying $\sum_{i=1}^{2}\left(\xi_{i}+\tau_{i}\right)=1$ and $\eta =\sum_{i=1}^{2}\left(\eta_{i}+\kappa_{i}\right)$. We assume *ξ*_*i*_ = 0.3 and *τ*_*i*_ = 0.2 to satisfy *ξ*_*i*_ > *τ*_*i*_ for *i*, *j* = 1, 2. Demographic parameters, such as the human birth rate (*μ*_*hb*_) and death rate (*μ*_*hd*_) on Jeju Island, were obtained from Statistics Korea [30]. These climate-independent parameters are listed in Table 4.

**Table 2:** Reports on yearly cases of dengue fever from 2001–2016 (unit: cases per year).

Year	2001	2002	2003	2004	2005	2006	2007	2008
Cases	6	9	14	16	34	35	97	51
Year	2009	2010	2011	2012	2013	2014	2015	2016
Cases	59	125	72	149	252	154	255	313

**Table 3:** Reports on monthly cases of dengue fever from 2012–2016 (unit: cases per month).

Year	Total	Jan	Feb	Mar	Apr	May	Jun	Jul	Aug	Sep	Oct	Nov	Dec
2012	149	4	7	5	6	3	9	20	26	24	18	16	11
2013	252	14	13	8	7	10	15	37	58	34	33	14	9
2014	165	13	8	5	6	10	14	32	26	11	18	17	5
2015	255	11	12	15	10	15	10	22	36	24	41	39	20
2016	313	32	36	32	23	18	22	39	42	24	13	18	14
Avr.	226.8	14.8	15.2	13	10.4	11.2	14	30	37.6	23.4	24.6	20.8	11.8

**Table 4:** Descriptions and values of parameters.

Symbol	Description	Value	Reference
*ν*	Vertical infection rate of *Aedes albopictus* mosquitoes	0.004	[33]
1/*α*	Latent period for human (day)	5	[34]
1/*γ*	Infectious period for human (day)	7	[11, 33, 35]
1/*α*_*i*_	Latent period for human with strain *i* (day)	5	[34]
1/*γ*_*i*_	Infectious period for human with strain *i* (day)	7	[11, 33, 35]
*ϕ*	Effect of antibody-dependent enhancement	1.5	[36]
*f*	Disease-induced mortality rate	0.005	[37]
*μ*_*hb*_	Human birth rate (*day*^−1^)	0.000020	[30]
*μ*_*hd*_	Human death rate (*day*^−1^)	0.000022	[30]
*N*_*v*_(0)	Initial number of mosquitoes	676000×2	[11, 30]
*N*_*h*_(0)	Initial number of human	676000	[30]
*x*_1_	Mosquito-to-human transmission probability	0.3841	estimated
*x*_2_	Human-to-mosquito transmission probability	1	estimated
*θ*	Insecticide control rate for vectors (*day*^−1^)	0-0.02	
*b*	Biting rate (*day*^−1^)	**	[38]
*b*_*h*_	Probability of transmission of the virus per bite (v→h) (*day*^−1^)	**	[39]
*b*_*m*_	Probability of transmission of the virus per bite (h→v) (*day*^−1^)	**	[39]
*μ*_*l*_	Mortality rates of the larvae (*day*^−1^)	**	[15]
*μ*_*v*_	Mortality rates of the mosquitoes (*day*^−1^)	**	[18]
*ω*	Pre-adult maturation rate (*day*^−1^)	**	[18]
*ε*	Virus incubation rate (*day*^−1^)	**	[40]
*β*_*vh*_	Transmissible rate (v→h) (*day*^−1^)	*x*_1_*bb*_*h*_	[39]
*β*_*hv*_	Transmissible rate (h→v) (*day*^−1^)	*x*_2_*bb*_*m*_	[39]
*δ*	Number of new recruits in the larvae stage (*day*^−1^)	*μ*_*v*_ *N*_*v*_ + *μ*_*l*_ *N*_*e*_	[33]
*η*	New infection rate by immigration (*day*^−1^)	**	[5]
*η*_*i*_	New primary infection rate by immigration for strain *i* (*day*^−1^)	**	[5]
*κ*_*i*_	New secondary infection rate by immigration for strain *i* (*day*^−1^)	**	[5]

(** refer to the time-dependent parameters estimated in the climate-dependent parameters section. The vector control rate *θ* refers to increase in the death rate of mosquitoes as *μ*_*v*_(1 + *θ*).)

#### Climate-dependent parameters

As described in the introduction, it is important to include more realistic parameters for dengue transmission. We incorporate the following temperature-dependent parameters: (1) *b*, the biting rate of an *Aedes* mosquito; (2) *b*_*m*_, the probability of infection from human to mosquito per bite; and (3) *b*_*h*_, the probability of infection from mosquito to human per bite. We include mosquito life cycle parameters, such as (4) *μ*_*v*_, the mortality rate and (5) *ω*, the pre-adult maturation rate. The biting rate *b* is described by a Brière function function [38]. Other parameter functions have been described over a temperature range of 10°*C* ≤ *T* ≤ 33°*C* in [18]. However, in Korea the average temperature in the winter is much lower than 10°*C*, and thus we estimate the parameter functions over a wider range of temperatures *T*. We also include (6) *ε*, the virus incubation rate, which is estimated from experimental data on the extrinsic incubation period for the range 13°C–35°C [40]; and (7) *μ*_*l*_, the larva mortality rate, which is related to the precipitation [15]. The expressions of these parameter functions are as follows:

1. The biting rate *b* is
  $$\begin{array}{c} b\left(T\right)=\{\begin{array}{cc} 0.000202T\left(T-13.35\right)\sqrt{40.08-T} & \left(13.35{}^{\circ}C\leq T\leq 40.08{}^{\circ}C\right)\\ 0 & \left(T<13.35{}^{\circ}C,T>40.08{}^{\circ}C\right) \end{array} \end{array}$$
2. The probability *b*_*h*_ of infection from mosquito to human per bite is
  $$\begin{array}{c} b_{h}\left(T\right)=\{\begin{array}{cc} 0.001044T\left(T-12.286\right)\sqrt{32.461-T} & \left(12.286{}^{\circ}C\leq T\leq 32.461{}^{\circ}C\right)\\ 0 & \left(T<12.286{}^{\circ}C,T>32.461{}^{\circ}C\right) \end{array} \end{array}$$
3. The probability *b*_*m*_ of infection from human to mosquito per bite is
  $$\begin{array}{c} b_{m}\left(T\right)=\{\begin{array}{cc} -0.9037+0.0729T & \left(12.4{}^{\circ}C\leq T<26.1{}^{\circ}C\right)\\ 1 & \left(26.1{}^{\circ}C\leq T\leq 32.5{}^{\circ}C\right)\\ 0 & \left(T<12.4{}^{\circ}C,T>32.5{}^{\circ}C\right) \end{array} \end{array}$$
4. Mortality rate *μ*_*v*_ of the adult mosquito is
  $$\begin{array}{c} \mu_{v}\left(T\right)=8.692\times 10^{-1}-1.590\times 10^{-1}T+1.116\times 10^{-2}T^{2}-3.408\times 10^{-4}T^{3}+3.809\times 10^{-6}T^{4} \end{array}$$
5. The pre-adult maturation rate *ω* is
  $$\begin{array}{ccc} \omega (T) & = & 0.1310-0.05723T+0.01164T^{2}-0.001341T^{3}+0.8723\times 10^{-4}T^{4}\\ & & -0.3017\times 10^{-5}T^{5}+0.5153\times 10^{-7}T^{6}+0.342\times 10^{-6}T^{7} \end{array}$$
  Note that *ω* is zero for *T* ≤10°C, because a larva cannot develop into a mosquito in this temperature range [18].
6. The virus incubation rate *ε* is
  $$\begin{array}{ccc} \varepsilon (T) & = & -1.678+0.344T-2.422\times 10^{-2}T^{2}+7.252\times 10^{-4}T^{3}-7.713\times 10^{-6}T^{4} \end{array}$$
7. The mortality rate *μ*_*l*_ of a larva is
  $$\begin{array}{c} \mu_{l}=\mu_{0}(1+\frac{N_{e}}{k_{l}}) \end{array}$$
  Here, *μ*_0_ = 0.08 is the minimum mortality rate and *k*_*l*_ = *k*_0_(*P*_*norm*_ + 1), where *k*_0_ = 250, 000 is the standard carry capacity in the environment for larvae, and *P*_*norm*_, whose value is between 0 and 1, is the normalized value of the amount of rainfall summed over the prior two-week period [15].

**Fig 4:**
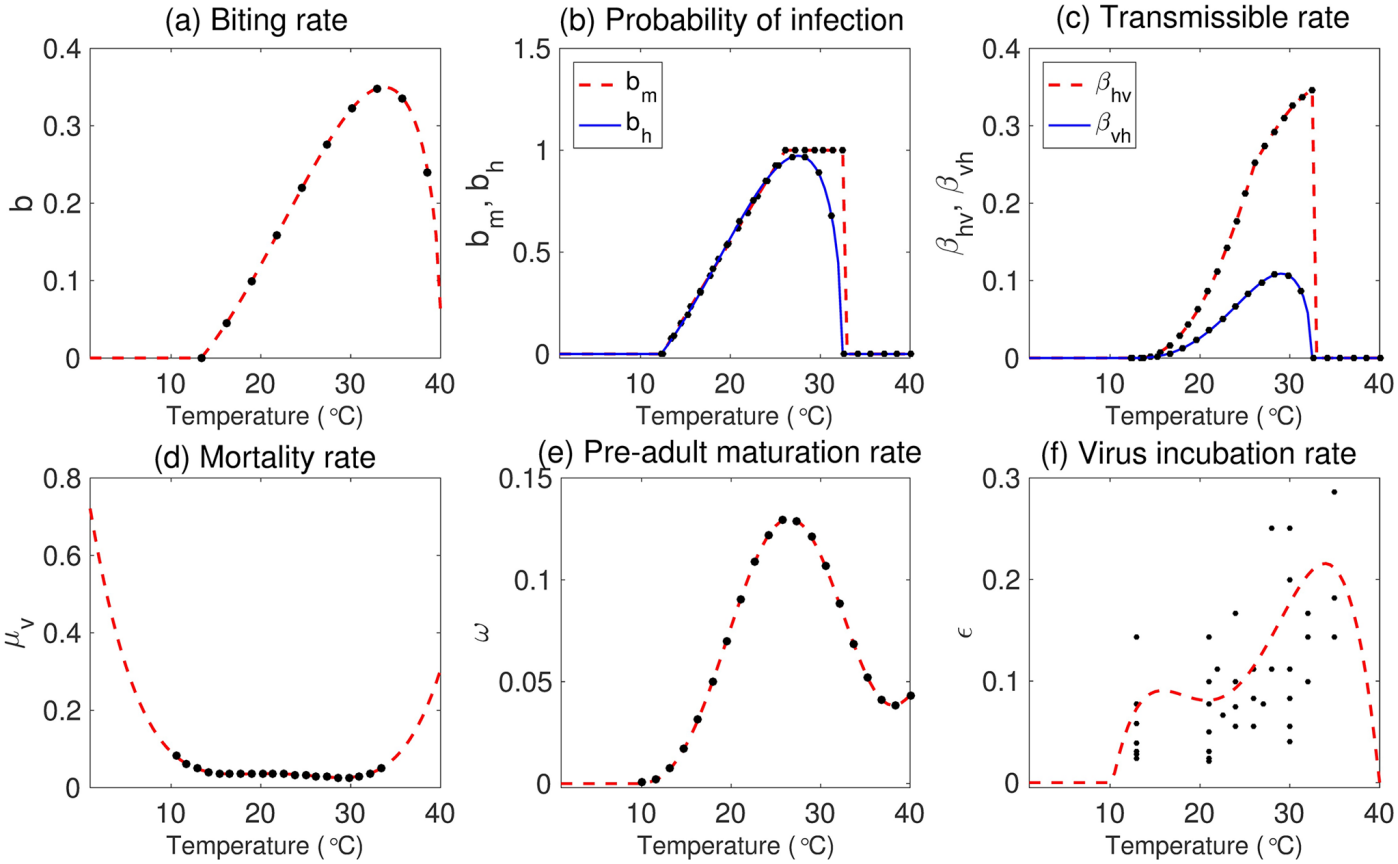
Temperature-dependent entomological parameters are displayed for various temperatures within the range 0°*C* to 40°*C*. In (a)—(e), red dashed and blue solid curves represent the extended parameters for a wider temperature range, and the black dots are values of fitting functions over the given temperature range. In (f), the red dashed curve represents the equation fitted using the black dotted experimental data.

In Fig 4, the temperature-dependent functions are displayed as black dots, and the extended functions fitted over a wider range of temperatures are displayed as red or blue dashed curves. Transmission rates are defined as *β*_*vh*_ = *x*_1_*bb*_*h*_ and *β*_*hv*_ = *x*_2_*bb*_*m*_, with transmission probabilities *x*_1_ and *x*_2_, respectively. The transmission probabilities *x*_1_ = 0.3841 and *x*_2_ = 1 are estimated from the 2014 dengue cases in Taiwan [41, 42] (see Fig 4(c), and refer to Section B in S1 Appendix for a detailed estimation). South Korea is located in East Asia, and Jeju Island experiences warmer and milder weather than other parts of South Korea. Hence, we assume that Jeju Island could have a similar climate to Taiwan over the next few decades. This is one reason that we used Taiwan dengue cases to estimate the transmission probabilities in our model.

To estimate the virus incubation rate *ε*, we employ a 4^*th*^-order polynomial function to fit the data with a zero value for the range *T* ≤ 10.3°C. Fig 4(f) illustrates the virus incubation rate depending on the temperature.

In the winter season, especially when the temperature is below 10 °*C*, the biting and transmissible rates are almost zero, and the mortality rate of adult mosquitoes is high. Thus, during this time period (below 10 °*C*), virus transmission rarely occurs between vectors and humans, although infected humans can still be present owing to infected international travelers returning from outside of South Korea.

## Results

The simulation results predict the climate-dependent behavior of dengue outbreaks for human and mosquito populations on Jeju Island, Korea. Climate changes are estimated for the RCP 2.6, RCP 4.5, RCP 6.0, and RCP 8.5 climate change scenarios. The total population size of the island is 676,000, as estimated from [30]. The birth and death rates per day are 0.000020 and 0.000022, respectively [30]. The initial human and mosquito population sizes are given by *N*_*h*_ = 676000 and *N*_*v*_ = 676000 × 2, respectively (concerning the results on different values of *N*_*v*_(0)/*N*_*h*_(0), refer to Section C in S1 Appendix). We assume that the initial numbers of infected mosquitoes and humans are zero so that the first infection is initiated by an inflow of infected international travelers.

### Dengue transmission dynamics based on RCP scenarios

**Fig 5:**
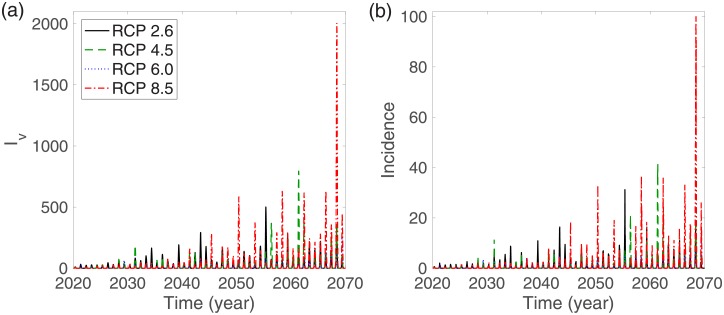
Single-strain model: (a) infectious mosquitoes and (b) incidence of humans are displayed over 50 years based on RCP 2.6 (black solid line), RCP 4.5 (green dashed line), RCP 6.0 (blue dotted line), and RCP 8.5 (red dash-dotted line). The initial conditions are set to *I*_*h*_(0) = 0, *I*_*v*_(0) = 0, *N*_*h*_(0) = 676000, and *N*_*v*_(0) = 2 × 676000.

**Fig 6:**
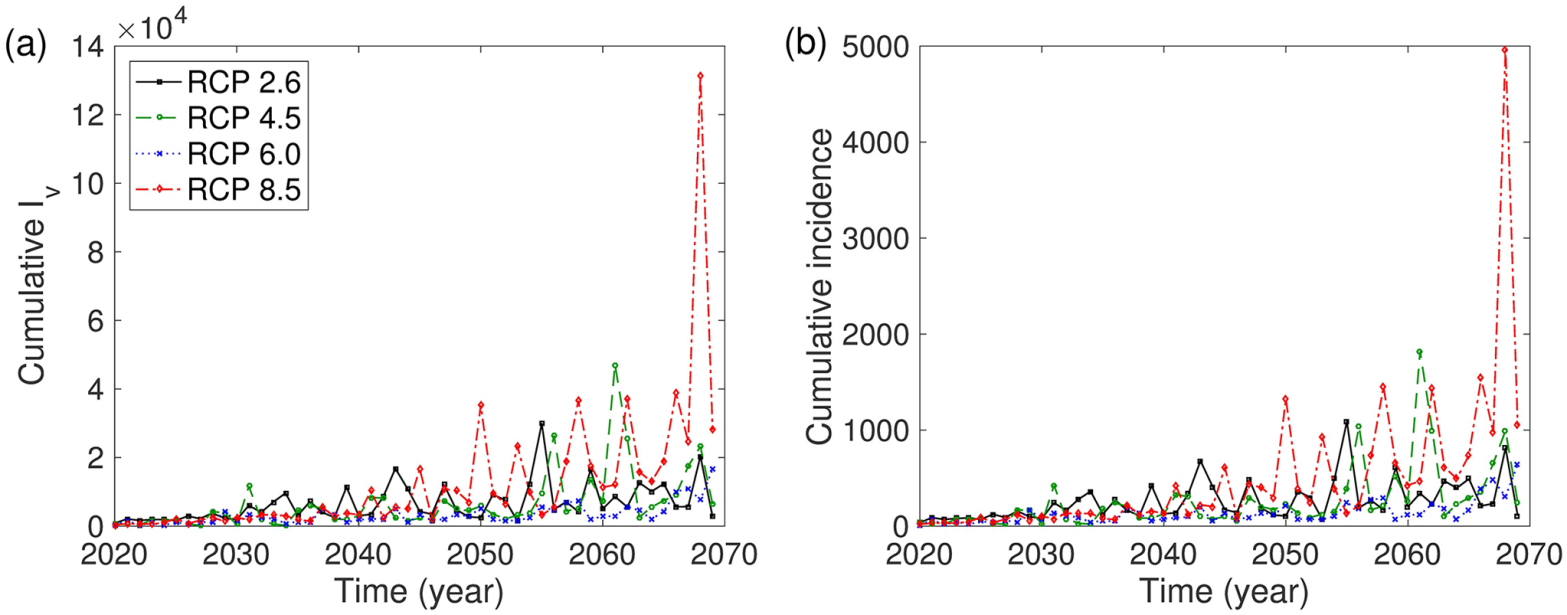
Single-strain model: (a) annual cumulative number of infectious mosquitoes and (b) annual cumulative incidence of humans are displayed over 50 years based on RCP 2.6 (black solid line), RCP 4.5 (green dashed line), RCP 6.0 (blue dotted line), and RCP 8.5 (red dash-dotted line). The initial conditions are set to *I*_*h*_(0) = 0, *I*_*v*_(0) = 0, *N*_*h*_(0) = 676000, and *N*_*v*_(0) = 2 × 676000.

In this section, we investigate the impact of various RCP scenarios on infectious mosquitoes and infectious humans for 50 years from June 1, 2020. Fig 5 shows the time evolution of infectious mosquitoes and the human incidence for the single-strain model. This demonstrates that dengue outbreaks for humans and mosquitoes exhibit recurrent seasonal patterns. Fig 6 depicts the annual cumulative number of infectious mosquitoes and the cumulative number of infectious humans over the 50 years in the single-strain model. This shows that higher-numbered RCP scenarios exhibit a tendency towards higher annual cumulative numbers of both infectious mosquitoes and humans on a long-term scale.

**Fig 7:**
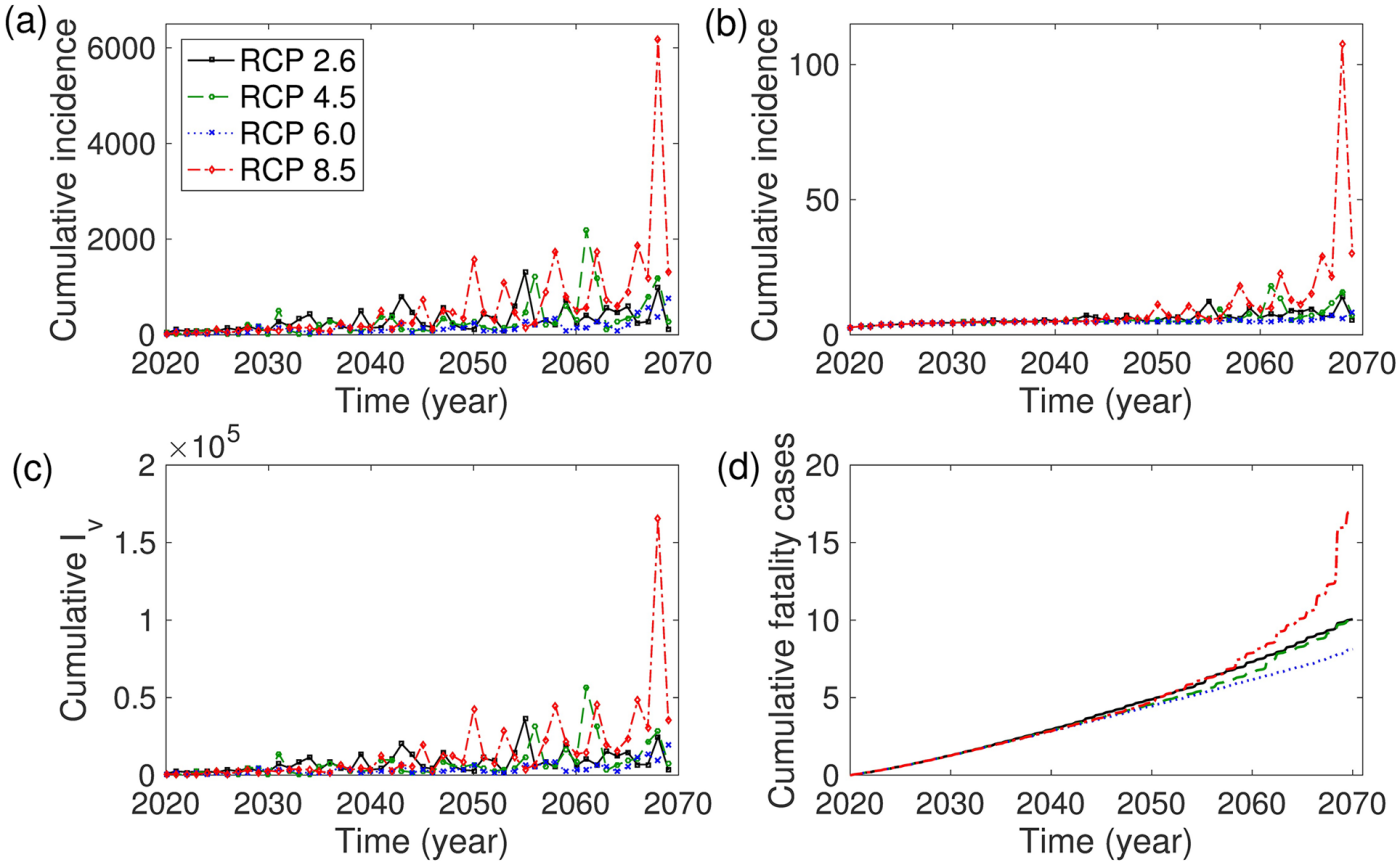
Two-strain model: (a) primary cumulative incidence of humans, (b) secondary cumulative incidence of humans, (c) cumulative number of infectious mosquitoes, and (d) cumulative fatality cases are displayed over 50 years based on RCP 2.6 (black), RCP 4.5 (green), RCP 6.0 (blue), and RCP 8.5 (red). The initial conditions are set to *I*_*v*1_(0) = *I*_*v*2_(0) = 0, *I*_*h*1_(0) = 0, *I*_*h*2_(0) = 0, *N*_*h*_(0) = 676000, and *N*_*v*_(0) = 2 × 676000.

Fig 7 shows the annual cumulative number of infectious mosquitoes and cumulative incidence of humans over the 50 years in the two-strain model. From the results, we see that there will be more infected mosquitoes and humans and fatalities in the RCP 8.5 scenario than the other scenarios on a long-term scale.

### Vectorial capacity and intensity under RCP scenarios

**Fig 8:**
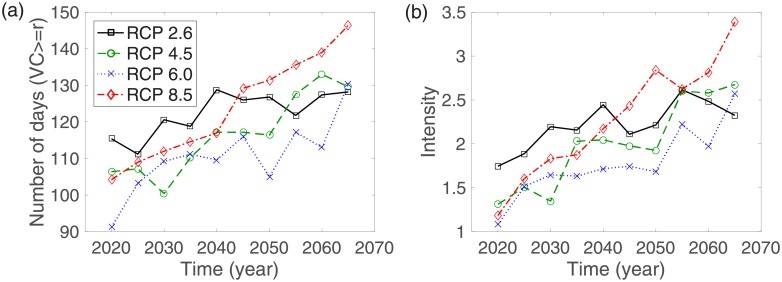
Two-strain model: (a) The five-year average of the numbers of days that have a higher VC than *γ* is displayed for each year. (b) The intensity is computed based on the average VC over the highest sequential three months for each five-year period.

The vectorial capacity (VC) is a quantity describing the epidemic potential of a vector-born disease, which represents the average number of potentially infectious contacts occurring for the vector population per infectious host per unit time [9, 43]. Liu-Helmersson et al. [44] investigated the effects of climate change on dengue transmission in Europe for the RCP scenarios 2.6 and 8.5. They provided an explicit form of the VC with six temperature-dependent parameters:

$$\begin{array}{c} VC=\frac{mb^{2}b_{h}b_{m}e^{-\frac{\mu_{v}}{\epsilon }}}{\mu_{v}} \end{array}$$ (4)

where $m=\frac{N_{v}}{N_{h}}$ is the female vector-to-human population ratio and other parameters are defined as in Table 4. Moreover, the basic reproduction number *R*_0_ of a vector-born disease can be written as

$$\begin{array}{c} R_{0}=\frac{mb^{2}b_{h}b_{m}e^{-\frac{\mu_{v}}{\epsilon }}}{\mu_{v}\gamma }=\frac{VC}{\gamma }, \end{array}$$ (5)

where *γ* is the recovery rate for humans [45]. Because an outbreak of the disease occurs when *R*_0_ > 1 and it becomes extinct when *R*_0_ < 1, the critical value of the VC for a dengue epidemic is VC* = *γ*. In order to observe changes in the potential for a dengue outbreak in our model, we consider two factors: the expected duration of an epidemic risk as the number of days for which *VC* > VC*, and the intensity of the VC defined as the average VC over the highest consecutive three months [44]. Fig 8 illustrates the average expected duration of epidemics and the average VC for five years for each RCP scenario. The expected duration of epidemics increases under all RCP scenarios, and increases by more than 30 days for the RCP 6.0 and 8.5 cases. Moreover, the intensity of the VC also increases in each RCP scenario, and it increases more than two-fold for the RCP 6.0 and 8.5 cases. This implies that the dengue epidemic risk increases if there is no control, and thus it is necessary to implement control strategies to reduce the risk of dengue outbreak.

### Correlation between cumulative dengue incidence and temperature

**Fig 9:**
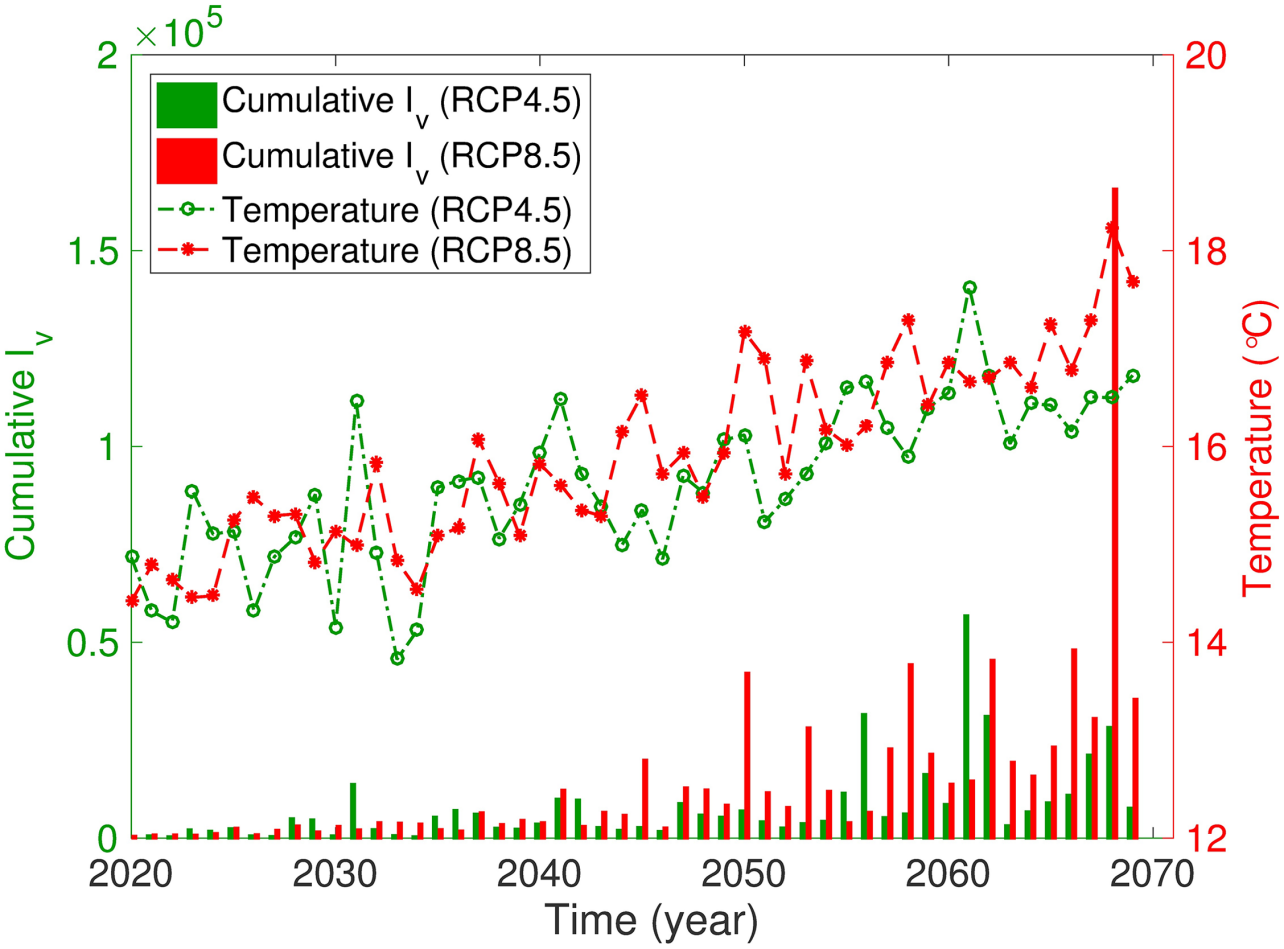
Two-strain model: Relationship between the cumulative number of infectious mosquitoes and temperature based on the RCP 4.5 (green) and RCP 8.5 (red) scenarios.

Fig 9 illustrates the relationship between the annual cumulative incidence of infected mosquitoes and the temperature based on the RCP 4.5 and 8.5 scenarios. Fig 9 implies that the annual cumulative number of infected mosquitoes and the temperature exhibit a strong relationship. To investigate this further, we compute the correlation between the annual cumulative number of infected mosquitoes and the temperature based on the two RCP scenarios. This correlation is commonly used to measure the strength of an association between two variables. As the value of the correlation coefficient approaches 1, the relationship between the two variables becomes stronger, and the directions of the relationship are sign + and sign −, which indicate a positive and negative relationship, respectively. We carry out two measures of non-parametric rank correlations: Spearman’s *ρ* and Kendall’s *τ* rank correlation coefficients [46]. Table 5 indicates that the temperature and cumulative number of infectious mosquitoes exhibit a significantly strong relationship.

**Table 5:** Correlation between cumulative number of infectious mosquitoes and RCP scenarios.

	Spearman		Kendall		Spearman		Kendall	
RCP	*ρ*	*p*	*τ*	*p*	*ρ*	*p*	*τ*	*p*
2.6	0.658	4.840 1E-7	0.473	1.33 1E-6	0.648	7.92 1E-7	0.466	1.86 1E-6
4.5	0.914	0	0.752	1.41 1E-14	0.924	0	0.765	4.90 1E-15
6.0	0.819	0	0.628	1.33 1E-10	0.821	0	0.631	1.06 1E-10
8.5	0.907	0	0.744	2.70 1E-14	0.912	0	0.755	1.08 1E-14
	(a) Single-strain model				(b) Two-strain model			

### The effects of travel restrictions and vector controls

Global warming combined with increases in the number of international travelers may trigger a potential risk for dengue outbreaks on Jeju Island. Here, we investigate the effects of two control strategies that should be implemented as countermeasures. The first control restricts infectious international travelers to Jeju Island, and the second reduces the vector population size by spraying insecticides. The first control can be achieved through multiplying the inflow rate of travelers by (1 − *u*). For the single-strain model, *η* is replaced by *η*(1 − *u*), where *u* is the reduction rate of a control strategy (0 ≤ *u* ≤ 1). Likewise, in the case of our two-strain model *η*_*i*_ and *κ*_*i*_ are replaced by *η*_*i*_(1 − *u*) and *κ*_*i*_(1 − *u*), respectively. Similarly, the insecticide control for vectors can be modeled by increasing the death rates of vectors as *μ*_*v*_(1 + *θ*) with a small positive number *θ* in both the single-strain and two-strain models.

**Fig 10:**
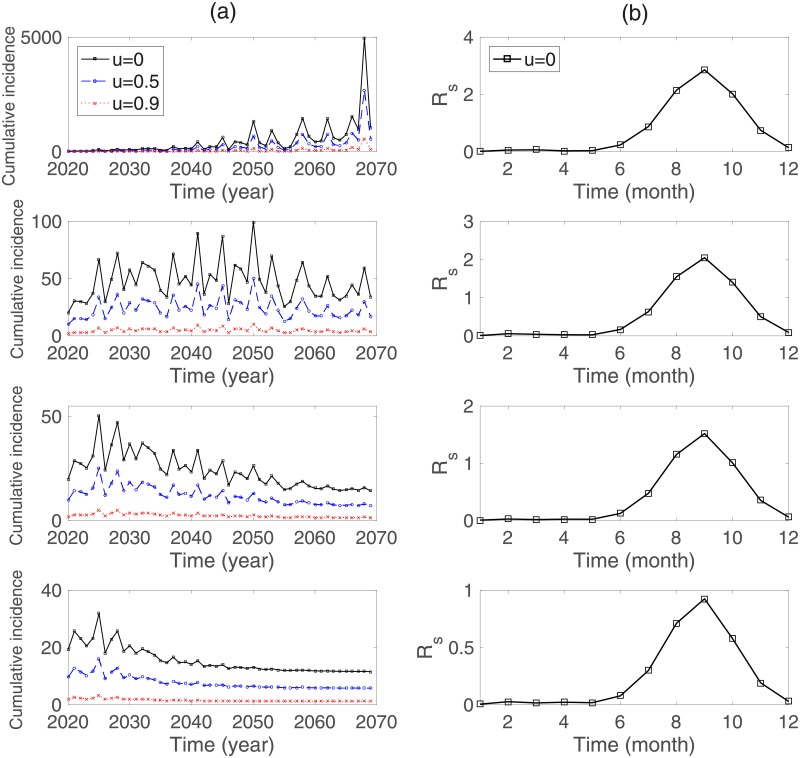
Single-strain model: (a) the annual cumulative incidence for humans and (b) the monthly averaged *R*_*s*_ are compared corresponding to *θ* = 0 (top), *θ* = 0.005 (second row), *θ* = 0.01 (third row), and *θ* = 0.02 (bottom). The climate data is based on the RCP 8.5 scenario.

**Fig 11:**
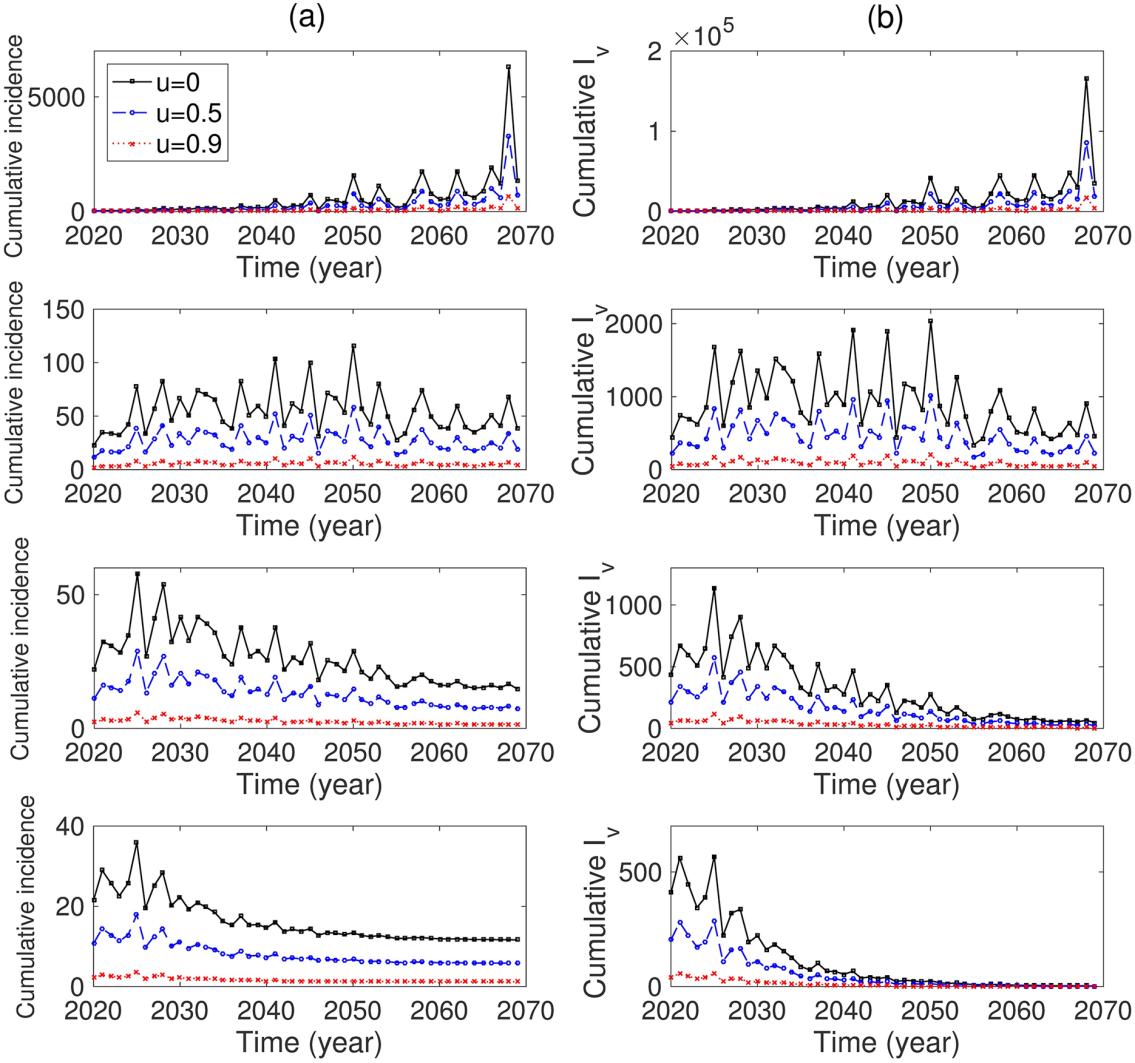
Two-strain model: (a) the annual cumulative incidence for humans and (b) cumulative number of infectious mosquitoes are compared corresponding to *θ* = 0 (top), *θ* = 0.005 (second row), *θ* = 0.01 (third row), and *θ* = 0.02 (bottom) over 50 years depending on *u* = 0 (black), 0.5 (blue), 0.9 (red) to control the inflow rate of travelers. The climate data is based on the RCP 8.5 scenario.

We explore the effects of travel restriction and vector controls on cumulative dengue cases for vectors and humans. First, the results for the single-strain model are presented in Fig 10. In the upper left subfigure of Fig 10, the annual cumulative incidences for vectors and humans are displayed using three different levels of travel restriction controls (*u* = 0: no control, *u* = 0.5 and *u* = 0.9: 50% and 90% reduction in the travel inflow rate, respectively). Clearly, the annual cumulative incidence decreases as *u* increases. Moreover, Fig 10 illustrates the results under combined control scenarios (travel restrictions and vector controls). Combined control is highly effective. Even a 0.5% increase in the vector control leads to a significant reduction in the annual cumulative incidence of humans (see the top panels with *θ* = 0.005). Moreover, the average *R*_*s*_ over all months decreases to under 1 as *θ* increases to 2%. Fig 11 and Table 6 display the effects of both controls on cumulative dengue cases in the two-strain model. These results indicate that the implementation of both intensive controls reduces the number of dengue cases dramatically. Therefore, travel restrictions alone are insufficient, and must be combined with vector controls.

**Table 6:** Two-strain model: The total number of cumulative incidences depending on control scenarios of dengue mosquitoes and infected travelers.

	*θ* = 0	*θ* = 0.005	*θ* = 0.01	*θ* = 0.02
*u* = 0	29138	2732	1311	815
*u* = 0.5	14917	1368	656	408
*u* = 0.9	3042	274	131	82

### Sensitivity analysis

We perform a sensitivity analysis on both the constant and temperature-dependent parameters. We define the normalized forward sensitivity index of the cumulative incidence as follows [47]:

$$\begin{array}{c} r_{p}^{\left(CI\right)}=\frac{\partial (CI)}{\partial p}\times \frac{p}{\left(CI\right)}. \end{array}$$

**Fig 12:**
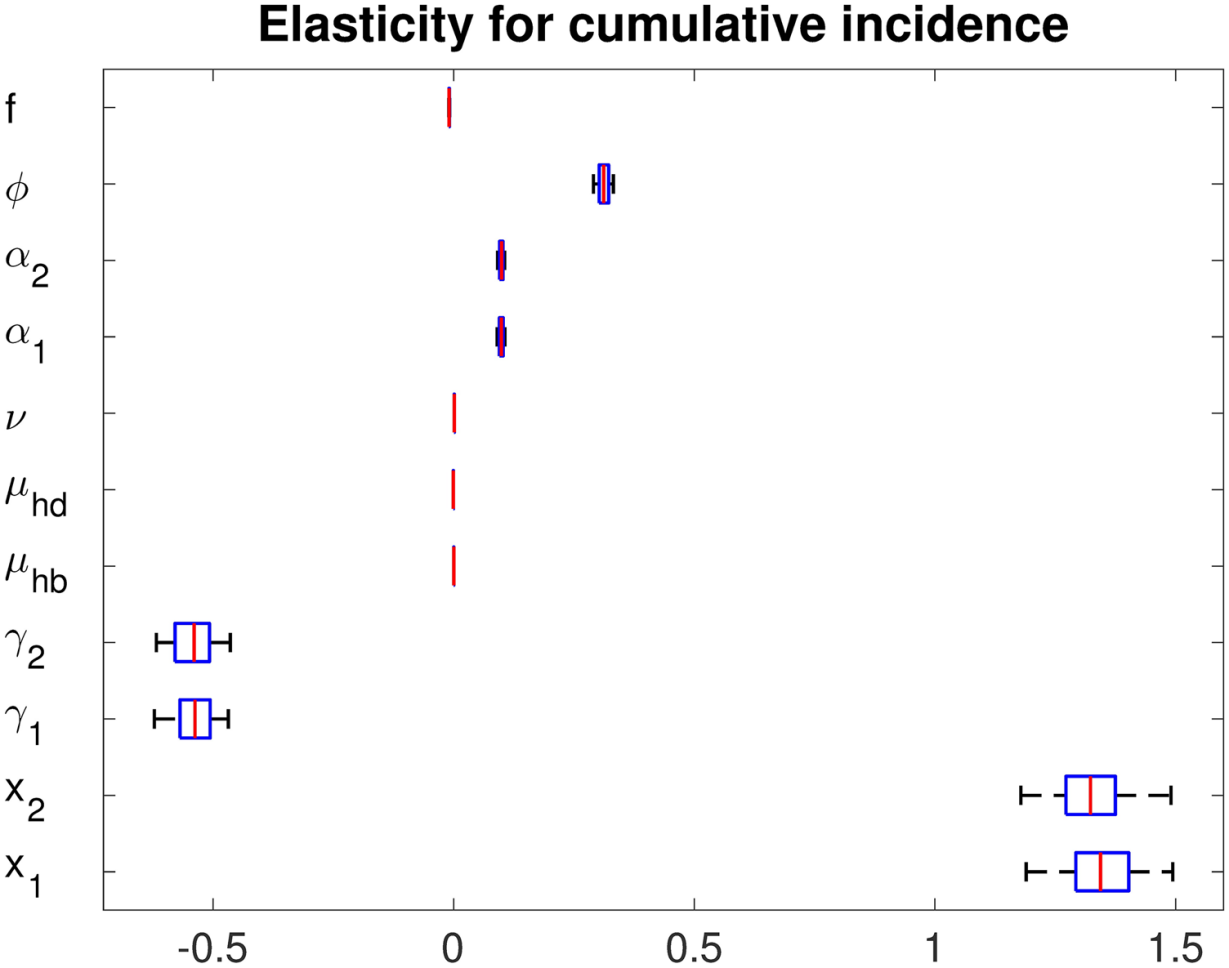
Elasticity of the cumulative incidence of humans: The two-strain model under the initial conditions *I*_*v*1_(0) = *I*_*v*2_(0) = 0, *I*_*h*1_(0) = 0, *I*_*h*2_(0) = 0, *N*_*h*_(0) = 676000, and *N*_*v*_(0) = 2×676000.

We randomly select 100 sets from a uniform distribution in the range of ± 10% of the baseline constant parameters in Table 4. Fig 12 shows the elasticity of the cumulative incidence (CI) with respect to the parameters. Setting June 1, 2020 as day 1, we compute the CI of infectious humans from day 1 to day 365 (one year). Fig 12 illustrates the elasticity of the cumulative incidence in the two-strain model. The cumulative incidence from day *t*_1_ to day *t*_2_ is defined as $\int_{t_{1}}^{t_{2}}(\alpha_{1}E_{h1}\left(t\right)+\alpha_{2}E_{h2}\left(t\right)+\alpha_{1}E_{h21}\left(t\right)+\alpha_{2}E_{h12}\left(t\right))dt$, where *t*_1_ = 1, *t*_2_ = 365. Here, *α*_*i*_ and *ϕ* are weakly positive influential parameters. Moreover, *γ*_*i*_ exerts the strongest negative influence on the CI among all the constant parameters. If *γ*_*i*_ is relatively increased by 20%, then the CI decreases by about 10%.

#### Sensitivity analysis of temperature-dependent parameters

**Fig 13:**
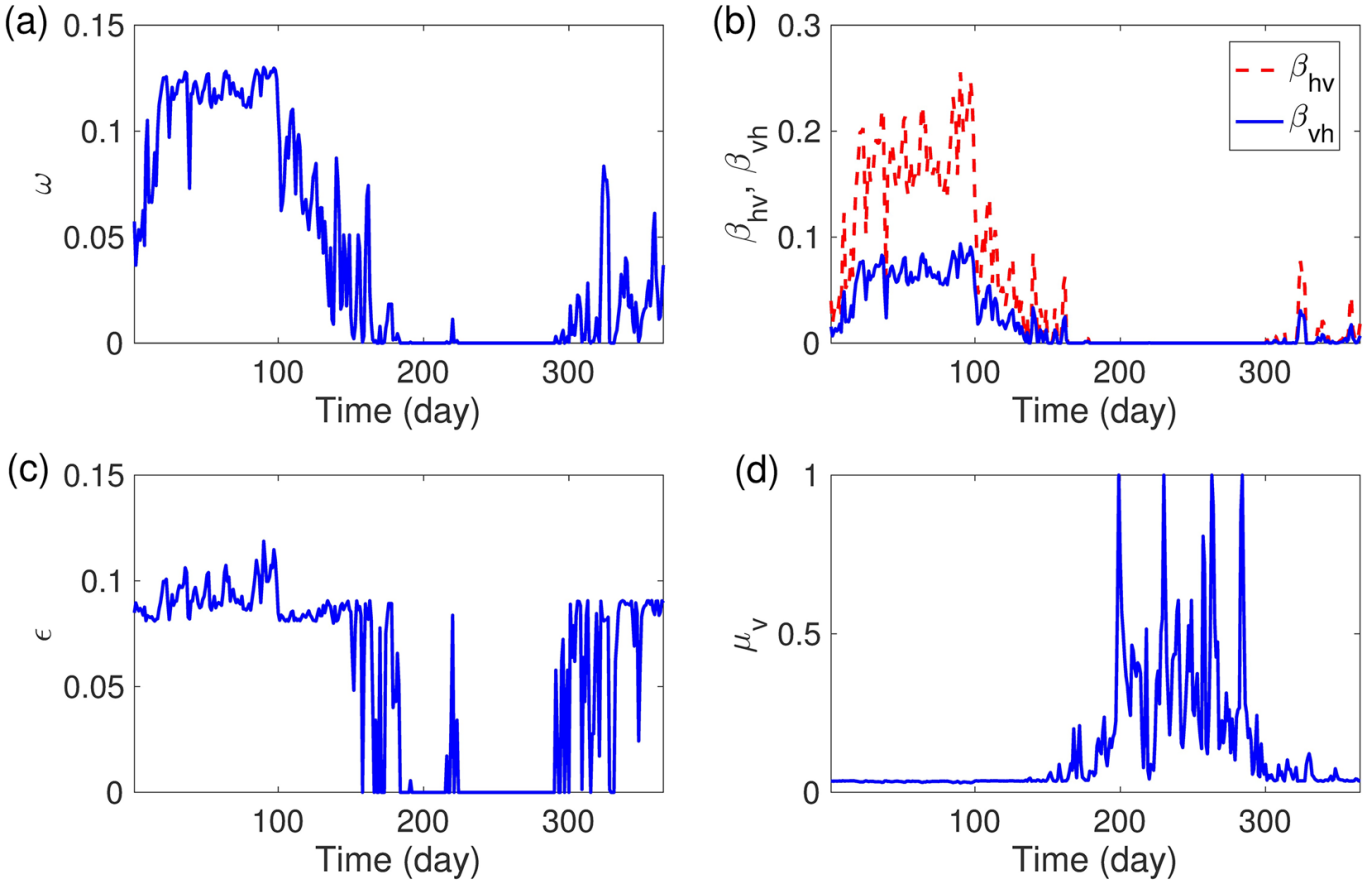
Daily temperature-dependent parameters are displayed over time based on the RCP 8.5 scenario starting on June 1, 2020. (a) Pre-adult maturation rate (*ω*), (b) transmissible rate (*β*_*hv*_, *β*_*hv*_), (c) virus incubation rate (*ε*), and (d) mortality rate (*μ*_*v*_).

We investigate the effects of temperature-dependent parameters on the seasonal reproduction number (*R*_*s*_). Fig 13 illustrates the changes in the temperature-dependent parameters under the RCP 8.5 scenario over 365 days beginning June 1, 2020. The maturation rate (*ω*), virus incubation rate (*ε*), and transmissible rates (*β*_*hv*_ and *β*_*vh*_) are maintained at high levels between day 1 and day 100, during the summer season. On the other hand, the mortality rate of mosquitoes (*μ*_*v*_) is close to zero during this time interval.

**Fig 14:**
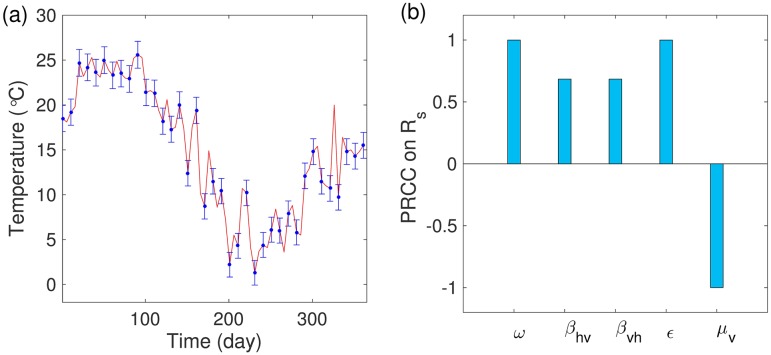
Random sampling: (a) Daily temperature based on RCP 8.5 (red solid) and the range of sampling for each temperature (blue line). (b) Partial rank correlation coefficients on *R*_*s*_ at day 150.

Fig 14(a) shows the daily temperature (°*C*) for RCP 8.5 and the range of a random sampling for each temperature. Fig 14(b) investigates the relationships between the parameters on *R*_*s*_ at a specific time point. We clearly observe that the mortality rate is a negative parameter, but the other parameters are positive parameters on *R*_*s*_ at day 150.

## Discussion

Recently, the climate of South Korea has changed from a warm temperate climate to a subtropical climate [48, 49], and this will make South Korea environmentally suited to the life cycles of mosquitos in the near future [50, 51]. In particular, dengue fever is predicted to be one of the most likely infectious diseases to threaten public health in Korea [6]. Recently, Japan, which is a neighboring country of Korea, has suffered an unexpected autochthonous dengue outbreak [52]. In the case of Korea, no indigenous dengue case has occurred so far, and all cases of infection have been diagnosed in travelers returning from endemic countries [5, 7]. However, it has been reported that the imported dengue cases in Korea and Japan exhibit a similar pattern [7], and the climate change and growing tendency in dengue cases for overseas travelers may trigger an autochthonous outbreak in South Korea [6, 7]. To the best of our knowledge, there has been no prior modeling study concerning the risk analysis for autochthonous dengue outbreaks associated with climate change in South Korea.

In order to investigate the risk of autochthonous dengue outbreaks in South Korea, we first constructed dengue transmission models for primary and secondary infections with climate-dependent parameters. These climate-dependent parameters were estimated from previous studies (experimental data and actual dengue cases). To estimate the transmission probabilities between mosquitoes and humans, we used dengue incidence data from Taiwan, because there have not yet been any indigenous dengue cases in Korea. The transmission probabilities could be estimated more accurately if indigenous dengue cases in Korea occur in the future.

Most of the modeling studies on dengue transmission associated with climate factors have focused on investigating the effects of temperature on the disease transmission [8–11, 18, 19, 38]. In this work, we incorporated the effects of rainfall as well as the temperature into our models. In particular, mosquito larvae inhabit bodies of water such as rivers, lakes, ponds, and swamps, and their mortality rate depends strongly on the amount of rainfall [15]. Thus, in our models we represented the larval mortality rate as a function of the amount of rainfall, so that the rainfall can affect the dynamics of dengue transmission.

Our study focused on Jeju Island, which has a warmer climate than other parts of South Korea. Thus, Jeju Island has a higher chance of having a favorable climate for *Aedes* mosquitoes [26, 51]. RCP scenario-based climate data for all city areas in Korea are currently provided by the Korea Meteorological Administration (KMA), and using these data the modeling approach presented in this paper could also be used to investigate the potential risks of dengue outbreak associated with climate change in other areas, such as subtropical southern city areas in Korea other than Jeju. Recent research has shown that over 20% of mosquitoes collected in South Korea are *Aedes* mosquitoes, and in particular the proportions of *Aedes* mosquitoes in park or hill areas are higher than those in dwelling areas [53]. Thus, one possible application of our modeling approach and a direction for our future work is motivated by the recent domestic dengue outbreak in Yoyogi Park, Tokyo, Japan [54]. This would consist of developing a two-patch model of dengue transmission [17] for dengue outbreaks between park/hill and dwelling areas in big cities such as Seoul or Busan in Korea.

Using the developed models, we illustrated the impact of climate change on the dynamics of dengue transmission in Jeju Island under various RCP scenarios provided by the KMA. Based on the RCP scenarios, the potential risk of dengue outbreak was assessed via the vectorial capacity (VC) over the next 50 years. We found that the intensity of the VC increases for higher RCP scenarios, which implies that the increase in average temperature owing to climate change may trigger a major dengue outbreak in Korea. It was also observed that the cumulative incidence of dengue mosquitoes and the temperature based on RCP scenarios exhibit a strong positive correlation. In particular, in case of RCP 8.5 a gradual increase in the temperature was predicted over the next 50 years, and massive dengue outbreaks may occur if adequate controls are not implemented. Concerning the controls of dengue transmission, we investigated the effects of controls for adult mosquitoes and infected travelers. If the cost-effectiveness of the two controls can be computed in future work, this would be of further help to the disease prevention authorities in implementing timely and effective control measures.

## Conclusion

In this paper, we developed dengue transmission models for primary and secondary infections with climate-dependent parameters to incorporate global warming effects into vector dynamics. We explored the impact of climate change on dengue transmission dynamics under Representative Concentration Pathway (RCP) scenarios. Moreover, we assessed the potential risks for dengue outbreaks via vectorial capacity and intensity, and we derived a formula for the seasonal reproduction number *R*_*s*_, which can be useful for analyzing the effects of climate change on the dengue transmission dynamics. Furthermore, we investigated the effects of controls by modifying two important factors of dengue transmission: the inflow rate of international travelers into Korea and the death rate of dengue mosquitoes. Controlling the inflow rate of infected international travelers is the main current control policy implemented by Korean governmental agents. However, controlling dengue mosquitoes in combination with the control of the inflow rate is highly effective. The results suggest that governmental agents should increase their efforts and budgets for controlling *Aedes* mosquitoes as well as infected humans, to reduce the risk of a dengue outbreak in Korea in the near future owing to climate change.

## Supporting information

S1 Appendix Supplementary figures and appendices. (PDF)
