# Supplementary material for: Potential effects of climate change on dengue transmission dynamics in Korea
Source: PLoS One. 2018 Jun 28;13(6):e0199205. doi: 10.1371/journal.pone.0199205 (PMC6023222; doi:10.1371/journal.pone.0199205)
Supplement: S1 Appendix — (PDF) [file pone.0199205.s001.pdf]

Supplementary Figures and Appendices

A. Seasonal reproduction number

The seasonal reproduction number, R_s, is an alternative form of the basic reproduction number R_0 [1]. R_0 is calculated as the spectral radius of the next generation matrix at the disease free equilibrium [2]. In a similar way, we can derive R_s which includes time-dependent parameters. In computing R_s for a single-strain model and a two-strain model, the inflow rate of dengue cases imported via international travel is assumed to be zero.

Seasonal reproduction number R_s for the single-strain model

The system of ordinary differential equations for the single-strain model has the disease-free state $\mathbf{x_0} = (S_e, 0, S_v, 0, 0, S_h, 0, 0, 0)$ with η=0.

$$\begin{aligned}
\frac{dI_e}{dt} &= \delta\nu\frac{I_v}{N_v} - \omega I_e - \mu_l\left(1+\frac{N_e}{k_l}\right)I_e \\
\frac{dE_v}{dt} &= \beta_{hv}\frac{I_h}{N_h}S_v - \varepsilon E_v - \mu_v E_v \\
\frac{dI_v}{dt} &= \varepsilon E_v + \omega I_e - \mu_v I_v \\
\frac{dE_h}{dt} &= \beta_{vh}\frac{S_h}{N_h}I_v - \alpha E_h - \mu_{hd}E_h \\
\frac{dI_h}{dt} &= \alpha E_h - \gamma I_h - \mu_{hd} I_h
\end{aligned} \tag{1}$$

Let $\mathbf{x} = (I_e, E_v, I_v, E_h, I_h)^T$, the system (1) is rewritten as $\mathbf{x}' = \mathcal{F} - \mathcal{V}$.

$\mathcal{F}(\mathbf{x})$ represents all new infections. The net transition rates out of the corresponding compartment are represented by $\mathcal{V}(\mathbf{x})$, where

$$\mathcal{F}(\mathbf{x}) = \begin{pmatrix} \delta\nu\frac{I_v}{N_v} \\ \beta_{hv}\frac{I_h}{N_h}S_v \\ 0 \\ \beta_{vh}\frac{S_h}{N_h}I_v \\ 0 \end{pmatrix}, \mathcal{V}(\mathbf{x}) = \begin{pmatrix} \omega I_e + \mu_l\left(1+\frac{N_e}{k_l}\right)I_e \\ (\varepsilon+\mu_v)E_v \\ -\varepsilon E_v - \omega I_e + \mu_v I_v \\ (\alpha+\mu_{hd})E_h \\ -\alpha E_h + (\gamma+\mu_{hd})I_h \end{pmatrix}$$

F and V are 5× 5 matrices are given by $F = [\frac{\partial \mathcal{F}}{\partial x_j}(x_0)]$ and $V = [\frac{\partial \mathcal{V}}{\partial x_j}(x_0)]$

$$F = \begin{bmatrix} 0 & 0 & \delta\nu/N_v & 0 & 0 \\ 0 & 0 & 0 & 0 & \beta_{hv}S_v/N_h \\ 0 & 0 & 0 & 0 & 0 \\ 0 & 0 & \beta_{vh}S_h/N_h & 0 & 0 \\ 0 & 0 & 0 & 0 & 0 \end{bmatrix}$$

$$V = \begin{bmatrix} \omega + \mu_l\left(1+\frac{S_e}{k_l}\right) & 0 & 0 & 0 & 0 \\ 0 & (\varepsilon+\mu_v) & 0 & 0 & 0 \\ -\omega & -\varepsilon & \mu_v & 0 & 0 \\ 0 & 0 & 0 & (\alpha+\mu_{hd}) & 0 \\ 0 & 0 & 0 & -\alpha & (\gamma+\mu_{hd}) \end{bmatrix}$$

and

$$V^{-1} = \begin{bmatrix} \frac{k_l}{k_l(\mu_l+\omega)+\mu_l S_e} & 0 & 0 & 0 & 0 \\ 0 & (\varepsilon+\mu_v)^{-1} & 0 & 0 & 0 \\ \frac{k_l\omega}{\mu_v(k_l(\mu_l+\omega)+\mu_l S_e)} & \frac{\varepsilon}{\mu_v(\varepsilon+\mu_v)} & \mu_v{}^{-1} & 0 & 0 \\ 0 & 0 & 0 & (\alpha+\mu_{hd})^{-1} & 0 \\ 0 & 0 & 0 & \frac{\alpha}{(\alpha+\mu_{hd})(\mu_{hd}+\gamma)} & (\gamma+\mu_{hd})^{-1} \end{bmatrix}$$

FV^{-1} is the next generation matrix of the system for the single-strain model;

$$FV^{-1} = \begin{bmatrix} \frac{\delta\nu k_l\omega}{\mu_v N_v(k_l(\mu_l+\omega)+\mu_l S_e)} & \frac{\delta\varepsilon\nu}{\mu_v N_v(\varepsilon+\mu_v)} & \frac{\delta\nu}{\mu_v N_v} & 0 & 0 \\ 0 & 0 & 0 & \frac{\alpha\beta_{hv}S_v}{N_h(\mu_{hd}+\gamma)(\mu_{hd}+\alpha)} & \frac{\beta_{hv}S_v}{N_h(\mu_{hd}+\gamma)} \\ 0 & 0 & 0 & 0 & 0 \\ \frac{\beta_{vh}k_l\omega S_h}{\mu_v N_v(k_l(\mu_l+\omega)+\mu_l S_e)} & \frac{\beta_{vh}\varepsilon S_h}{\mu_v N_h(\varepsilon+\mu_v)} & \frac{\beta_{vh}S_h}{\mu_v N_h} & 0 & 0 \\ 0 & 0 & 0 & 0 & 0 \end{bmatrix}$$

Thus, the seasonal reproduction number of the system for the single-strain model at time t in the absence of the inflow rate of international travelers (i.e., $\eta = 0$) is given by the spectral radius of matrix FV^{-1} as follows;

$$R_s = \frac{A}{2} + \frac{1}{2}\sqrt{A^2 + 4\Lambda} \quad (2)$$

$$A = \frac{\delta(t)k_l(t)\omega(t)\nu}{\mu_v(t)N_v(t)(k_l(t)(\mu_l+\omega(t))+\mu_l S_e(t))}, \quad (3)$$

$$\Lambda = \frac{\alpha\beta_{hv}(t)\beta_{vh}(t)\varepsilon(t)S_h(t)S_v(t)}{(\alpha+\mu_{hd})\mu_v(t)(\varepsilon(t)+\mu_v(t))(\mu_{hd}+\gamma)N_h(t)^2}$$

Fig. A shows the relationship between the seasonal reproduction number, temperature and the number of infectious humans for six years in the single-strain model. Fig. A (a) compares the dengue incidence with temperature change. Fig A (b) shows that peaks in R_s and temperature occur almost at the same time.

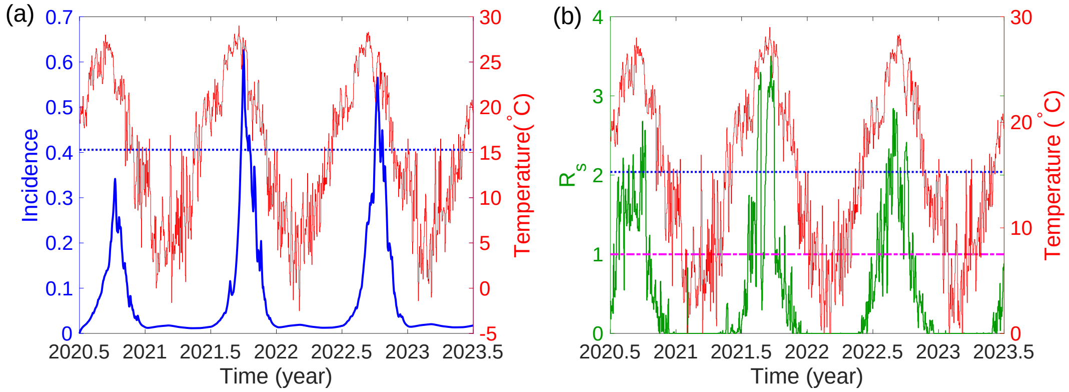

Fig A. (a) Incidence of humans (blue solid) and (b) The seasonal reproduction number R_s (green dashed) based on scenario RCP 8.5 (red dashed) during the three years. The initial condition is $I_h(0)$=0, $I_v(0)$=0, $N_h(0)$=676000, and $N_v(0)$=2×676000. The blue dotted horizontal line represents the average temperature (14.6°C) during the three years and magenta dash-dotted horizontal line represents R_s=1.

Seasonal reproduction number R_s for the two-strain model

When the inflow rate of dengue cases imported via international travel is absent, we consider the disease free state $\mathbf{x_0}$ consisting of a 21 × 1 zero vector except for S_e, S_v, S_h.

Vector

$$
\begin{aligned}
\frac{dI_{e1}}{dt} &= \delta\nu\frac{I_{v1}}{N_v} - \omega_1 I_{e1} - \mu_l\left(1+\frac{N_e}{k_l}\right)I_{e1} \\
\frac{dI_{e2}}{dt} &= \delta\nu\frac{I_{v2}}{N_v} - \omega_2 I_{e2} - \mu_l\left(1+\frac{N_e}{k_l}\right)I_{e2} \\
\frac{dE_{v1}}{dt} &= \beta_{hv}\left(\frac{I_{h1}+\phi I_{h21}}{N_h}\right)S_v - \varepsilon E_{v1} - \mu_v E_{v1} \\
\frac{dE_{v2}}{dt} &= \beta_{hv}\left(\frac{I_{h2}+\phi I_{h12}}{N_h}\right)S_v - \varepsilon E_{v2} - \mu_v E_{v2} \\
\frac{dI_{v1}}{dt} &= \omega_1 I_{e1} + \varepsilon E_{v1} - \mu_v I_{v1} \\
\frac{dI_{v2}}{dt} &= \omega_2 I_{e2} + \varepsilon E_{v2} - \mu_v I_{v2}
\end{aligned}
\tag{4}
$$

Host

$$
\begin{aligned}
\frac{dE_{h1}}{dt} &= S_h\left(\beta_{vh}\frac{I_{v1}}{N_h}\right) + \eta_1 S_h - (\alpha_1+\mu_{hd})E_{h1} \\
\frac{dE_{h2}}{dt} &= S_h\left(\beta_{vh}\frac{I_{v2}}{N_h}\right) + \eta_2 S_h - (\alpha_2+\mu_{hd})E_{h2} \\
\frac{dI_{h1}}{dt} &= \alpha_1 E_{h1} - \gamma_1 I_{h1} - \mu_{hd} I_{h1} \\
\frac{dI_{h2}}{dt} &= \alpha_2 E_{h2} - \gamma_2 I_{h2} - \mu_{hd} I_{h2} \\
\frac{dE_{h12}}{dt} &= R_{h1}\beta_{vh}\left(\frac{I_{v2}}{N_h}\right) + \kappa_2 S_h - \alpha_2 E_{h12} - \mu_{hd}E_{h12} \\
\frac{dE_{h21}}{dt} &= R_{h2}\beta_{vh}\left(\frac{I_{v1}}{N_h}\right) + \kappa_1 S_h - \alpha_1 E_{h21} - \mu_{hd}E_{h21} \\
\frac{dI_{h12}}{dt} &= \alpha_2 E_{h12} - (\gamma_2+\mu_{hd}+f)\,I_{h12} \\
\frac{dI_{h21}}{dt} &= \alpha_1 E_{h21} - (\gamma_1+\mu_{hd}+f)\,I_{h21}
\end{aligned}
\tag{5}
$$

Let $\mathbf{x} = (I_{ei}, E_{vi}, I_{vi}, E_{hi}, I_{hi})^T$, the system (4) and (5) are rewritten as $\mathbf{x}' = \mathcal{F} - \mathcal{V}$, where

$$\mathcal{F}(\mathbf{x}) = \begin{pmatrix} \delta\nu\frac{I_{v1}}{N_v} \\ \delta\nu\frac{I_{v2}}{N_v} \\ \beta_{hv}\left(\frac{I_{h1}+\phi I_{h21}}{N_h}\right)S_v \\ \beta_{hv}\left(\frac{I_{h2}+\phi I_{h12}}{N_h}\right)S_v \\ 0 \\ 0 \\ S_h\left(\beta_{vh}\frac{I_{v1}}{N_h}\right) \\ S_h\left(\beta_{vh}\frac{I_{v2}}{N_h}\right) \\ 0 \\ 0 \\ \beta_{vh}R_{h1}\frac{I_{v2}}{N_h} \\ \beta_{vh}R_{h2}\frac{I_{v1}}{N_h} \\ 0 \\ 0 \end{pmatrix}, \mathcal{V}(\mathbf{x}) = \begin{pmatrix} \omega_1 I_{e1} + \mu_l\left(1+\frac{N_e}{k_l}\right)I_{e1} \\ \omega_2 I_{e2} + \mu_l\left(1+\frac{N_e}{k_l}\right)I_{e2} \\ (\varepsilon+\mu_v)E_{v1} \\ (\varepsilon+\mu_v)E_{v2} \\ -\omega_1 I_{e1} - \varepsilon E_{v1} + \mu_v I_{v1} \\ -\omega_2 I_{e2} - \varepsilon E_{v2} + \mu_v I_{v2} \\ (\alpha_1+\mu_{hd})E_{h1} \\ (\alpha_2+\mu_{hd})E_{h2} \\ -\alpha_1 E_{h1} + (\gamma_1+\mu_{hd})I_{h1} \\ -\alpha_2 E_{h2} + (\gamma_2+\mu_{hd})I_{h2} \\ (\alpha_2+\mu_{hd})E_{h12} \\ (\alpha_1+\mu_{hd})E_{h21} \\ -\alpha_2 E_{h12} + (\gamma_2+\mu_{hd}+f)\,I_{h12} \\ -\alpha_1 E_{h21} + (\gamma_1+\mu_{hd}+f)\,I_{h21} \end{pmatrix}$$

The net transition rates of the corresponding compartment are represented by $\mathcal{V}(\mathbf{x})$, where F and V are $14\times$ 14 matrices at x_0 given by

$$F = \begin{bmatrix} 0&0&0&0&f_{1,5}&0&0&0&0&0&0&0&0&0 \\ 0&0&0&0&0&f_{2,6}&0&0&0&0&0&0&0&0 \\ 0&0&0&0&0&0&0&0&f_{3,9}&0&0&0&0&f_{3,14} \\ 0&0&0&0&0&0&0&0&0&f_{4,10}&0&0&f_{4,13}&0 \\ 0&0&0&0&0&0&0&0&0&0&0&0&0&0 \\ 0&0&0&0&0&0&0&0&0&0&0&0&0&0 \\ 0&0&0&0&f_{7,5}&0&0&0&0&0&0&0&0&0 \\ 0&0&0&0&0&f_{8,6}&0&0&0&0&0&0&0&0 \\ 0&0&0&0&0&0&0&0&0&0&0&0&0&0 \\ 0&0&0&0&0&0&0&0&0&0&0&0&0&0 \\ 0&0&0&0&0&0&0&0&0&0&0&0&0&0 \\ 0&0&0&0&0&0&0&0&0&0&0&0&0&0 \\ 0&0&0&0&0&0&0&0&0&0&0&0&0&0 \\ 0&0&0&0&0&0&0&0&0&0&0&0&0&0 \end{bmatrix}$$

$$\begin{array}{llll} f_{1,5} = \frac{\delta\nu}{N_v} & f_{2,6} = \frac{\delta\nu}{N_v} & f_{3,9} = \frac{\beta_{hv}S_v}{N_h} & f_{3,14} = \frac{\beta_{hv}\phi S_v}{N_h} \\ f_{4,10} = \frac{\beta_{hv}S_v}{N_h} & f_{4,13} = \frac{\beta_{hv}\phi S_v}{N_h} & f_{7,5} = \frac{\beta_{vh}S_h}{N_h} & f_{8,6} = \frac{\beta_{vh}S_h}{N_h} \end{array}$$

and

$$V =$$

$$\begin{bmatrix}
v_{1,1} & 0 & 0 & 0 & 0 & 0 & 0 & 0 & 0 & 0 & 0 & 0 & 0 & 0 \\
0 & v_{2,2} & 0 & 0 & 0 & 0 & 0 & 0 & 0 & 0 & 0 & 0 & 0 & 0 \\
0 & 0 & v_{3,3} & 0 & 0 & 0 & 0 & 0 & 0 & 0 & 0 & 0 & 0 & 0 \\
0 & 0 & 0 & v_{4,4} & 0 & 0 & 0 & 0 & 0 & 0 & 0 & 0 & 0 & 0 \\
v_{5,1} & 0 & v_{5,3} & 0 & v_{5,5} & 0 & 0 & 0 & 0 & 0 & 0 & 0 & 0 & 0 \\
0 & v_{6,2} & 0 & v_{6,4} & 0 & v_{6,6} & 0 & 0 & 0 & 0 & 0 & 0 & 0 & 0 \\
0 & 0 & 0 & 0 & 0 & 0 & v_{7,7} & 0 & 0 & 0 & 0 & 0 & 0 & 0 \\
0 & 0 & 0 & 0 & 0 & 0 & 0 & v_{8,8} & 0 & 0 & 0 & 0 & 0 & 0 \\
0 & 0 & 0 & 0 & 0 & 0 & v_{9,7} & 0 & v_{9,9} & 0 & 0 & 0 & 0 & 0 \\
0 & 0 & 0 & 0 & 0 & 0 & 0 & v_{10,8} & 0 & v_{10,10} & 0 & 0 & 0 & 0 \\
0 & 0 & 0 & 0 & 0 & 0 & 0 & 0 & 0 & 0 & v_{11,11} & 0 & 0 & 0 \\
0 & 0 & 0 & 0 & 0 & 0 & 0 & 0 & 0 & 0 & 0 & v_{12,12} & 0 & 0 \\
0 & 0 & 0 & 0 & 0 & 0 & 0 & 0 & 0 & 0 & v_{13,11} & 0 & v_{13,13} & 0 \\
0 & 0 & 0 & 0 & 0 & 0 & 0 & 0 & 0 & 0 & 0 & v_{14,12} & 0 & v_{14,14}
\end{bmatrix}$$

$$\begin{array}{cccc}
v_{1,1} = \omega_1 + \mu_l(1 + \frac{S_e}{k_l}) & v_{2,2} = \omega_2 + \mu_l(1 + \frac{S_e}{k_l}) & v_{3,3} = (\varepsilon + \mu_v) & v_{4,4} = (\varepsilon + \mu_v) \\
v_{5,5} = \mu_v & v_{6,6} = \mu_v & v_{7,7} = (\alpha_1 + \mu_{hd}) & v_{8,8} = (\alpha_2 + \mu_{hd}) \\
v_{9,9} = (\gamma_1 + \mu_{hd}) & v_{10,10} = (\gamma_2 + \mu_{hd}) & v_{11,11} = (\alpha_2 + \mu_{hd}) & v_{12,12} = (\alpha_1 + \mu_{hd}) \\
v_{13,13} = (\gamma_2 + \mu_{hd} + f) & v_{14,14} = (\gamma_1 + \mu_{hd} + f) & v_{5,1} = -\omega_1 & v_{5,3} = -\varepsilon \\
v_{6,2} = -\omega_2 & v_{6,4} = -\varepsilon & v_{9,7} = -\alpha_1 & v_{10,8} = -\alpha_2 \\
v_{13,11} = -\alpha_2 & v_{14,12} = -\alpha_1 & &
\end{array}$$

FV^{-1} is the next generation matrix of the system of ordinary differential equations for the two-strain model. Thus, the seasonal reproduction number of the system for the two-strain model in the absence of inflow of international travelers (i.e., $\eta_i = \kappa_i = 0$) is given by the spectral radius of matrix FV^{-1} as follows:

$$\begin{aligned}
R_s &= max(R_{s1}, R_{s2}) \\
R_{s1} &= \frac{A}{2} + \frac{1}{2}\sqrt{A^2 + 4\Lambda_1}, \quad R_{s2} = \frac{A}{2} + \frac{1}{2}\sqrt{A^2 + 4\Lambda_2} \\
\text{where} \quad A &= \frac{\delta(t)k_l(t)\omega_i(t)\nu}{\mu_v(t)N_v(t)(k_l(t)(\mu_l + \omega_i(t)) + \mu_l S_e(t))}, \\
\Lambda_i &= \frac{\alpha_i \beta_{hv}(t)\beta_{vh}(t)\varepsilon(t)S_h(t)S_v(t)}{(\alpha_i + \mu_{hd})\mu_v(t)(\varepsilon(t) + \mu_v(t))(\mu_{hd} + \gamma_i)N_h(t)^2}
\end{aligned}$$

B. Data fitting for x_1 and x_2 by using least square method

We compare the dengue incidence data in Taiwan with the results of numerical simulations (in the absence of imported dengue cases) to confirm the validity of parameter values in the model and determine x_1 and x_2 through the data fitting.

According to Centers for Disease Control of Taiwan, Taiwan experienced the large dengue fever outbreak in 2014 and a consecutive larger dengue fever outbreak in 2015, resulting in a total of 51579 cases. We focus on the 2014 dengue fever outbreak to fit our models to the data without

control because the control strategies have not been implemented before the dengue major outbreak in 2015. The vertical infection rate (ν) is 0.028 [3] and population size in Taiwan in 2014 was 23,434,000 with the births and deaths 210383 and 163929, respectively, from which birth rate (μ_{hb}) and death rate (μ_{hd}) per day are computed as 0.000025 and 0.000019, respectively.

In order to obtain the transmission probabilities x_1 and x_2 from data fitting, we carry out numerical simulation for the period from week 1 (2013.12.29 − 2013.1.4) to week 52 (2014.12.21 − 2014.12.27). We set the initial infected human as 7 dengue cases on week 1 and the mosquito population size is two times larger than human population size initially.

Data fitting procedure

Step 1. We generate the daily temperature and dengue incidence data. All weekly confirmed dengue cases are provided by Centers for Disease Control of Taiwan and there are 15,814 reported dengue cases during week 1 − week 52. Monthly temperature and precipitation are provided by Central Weather Bureau in Taiwan. Then, we generate the daily temperature and precipitation data and dengue incidence by using cubic spline interpolation.

Step 2. The daily incidence and weekly incidence are computed for single-strain model. The incidence at day t is defined by $\alpha E_h(t)$. The weekly incidence during seven days from day t_1 to day t_2 is computed as $\int_{t_1}^{t_2} \alpha E_h(t)dt$.

Step 3. We carry out data fitting with least squares. We use **LSQcurvefit** function of Matlab which implements data fitting with nonlinear least squares methods during the period from week 0 (2013.12.29 − 2013.1.4) to week 52 (2014.12.21 − 2014.12.27). As a result, we obtain $x_1 = 0.3841$ and $x_2 = 1$.

C. Simulation results about different values of $N_v(0)/N_h(0)$

Fig. B shows the annual cumulative number of infectious mosquitoes and cumulative number of infectious humans for 50 years in the single-strain model with different initial ratios $N_v(0)/N_h(0) = 1, 2, 3$. We observe that there are some quantitative differences between different ratios, but the qualitative dynamics are similar in all the cases.

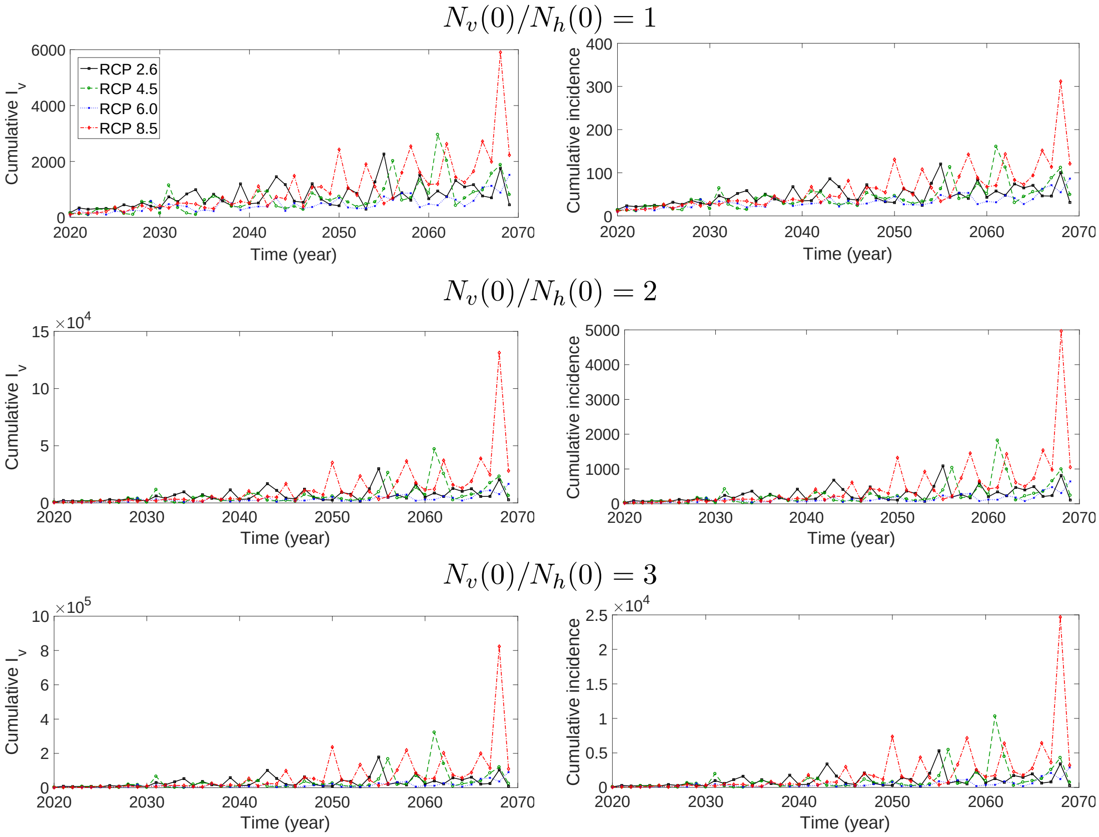

Fig B. Single-strain model with different initial values of N_V/N_h. Annual cumulative number of infectious mosquitoes (left figures) and annual cumulative incidence of humans (right figures) are displayed during 50 years based on RCP 2.6 (black), RCP 4.5 (green), RCP 6.0 (blue) and RCP 8.5 (red). The initial conditions are set to $I_v(0) = 0, I_h(0) = 0,\ N_h(0) = 676000$.

References

1. Kim J, Lee H, Lee C, Lee S. Assessment of optimal strategies in a two-patch dengue transmission model with seasonality. PLoS One. 2017;12(3):e0173673.
2. Brauer F, Castillo-Chavez C, Castillo-Chavez C. Mathematical models in population biology and epidemiology. vol. 40. Springer; 2001.
3. Adams B, Boots M. How important is vertical transmission in mosquitoes for the persistence of dengue? Insights from a mathematical model. Epidemics. 2010;2(1):1–10.
